# Characterization of Continuous Transcriptional Heterogeneity in High-Risk Blastemal-Type Wilms’ Tumors Using Unsupervised Machine Learning

**DOI:** 10.3390/ijms24043532

**Published:** 2023-02-09

**Authors:** Yaron Trink, Achia Urbach, Benjamin Dekel, Peter Hohenstein, Jacob Goldberger, Tomer Kalisky

**Affiliations:** 1 Faculty of Engineering and Bar-Ilan Institute of Nanotechnology and Advanced Materials (BINA), Bar-Ilan University, Ramat Gan 5290002, Israel; 2 The Mina and Everard Goodman Faculty of Life Sciences, Bar-Ilan University, Ramat Gan 5290002, Israel; 3 Pediatric Stem Cell Research Institute and Division of Pediatric Nephrology, Edmond and Lily Safra Children’s Hospital, Sheba Medical Center, Tel-Hashomer 5262000, Israel; 4 Department of Human Genetics, Leiden University Medical Center, 2300 RC Leiden, The Netherlands

**Keywords:** Wilms’ tumors, pareto task inference, topic modeling, cellular deconvolution

## Abstract

Wilms’ tumors are pediatric malignancies that are thought to arise from faulty kidney development. They contain a wide range of poorly differentiated cell states resembling various distorted developmental stages of the fetal kidney, and as a result, differ between patients in a continuous manner that is not well understood. Here, we used three computational approaches to characterize this continuous heterogeneity in high-risk blastemal-type Wilms’ tumors. Using Pareto task inference, we show that the tumors form a triangle-shaped continuum in latent space that is bounded by three tumor archetypes with “stromal”, “blastemal”, and “epithelial” characteristics, which resemble the un-induced mesenchyme, the cap mesenchyme, and early epithelial structures of the fetal kidney. By fitting a generative probabilistic “grade of membership” model, we show that each tumor can be represented as a unique mixture of three hidden “topics” with blastemal, stromal, and epithelial characteristics. Likewise, cellular deconvolution allows us to represent each tumor in the continuum as a unique combination of fetal kidney-like cell states. These results highlight the relationship between Wilms’ tumors and kidney development, and we anticipate that they will pave the way for more quantitative strategies for tumor stratification and classification.

## 1. Introduction

Wilms’ tumor, also known as nephroblastoma, is a pediatric malignancy that develops in children under age five. It is the most common type of kidney cancer in children, with approximately 600 new cases diagnosed each year in the United States [1]. Wilms’ tumors are highly heterogeneous and contain varying proportions of cells resembling renal progenitors and poorly differentiated structures. Therefore, it is thought that they arise from genetic and epigenetic distortions during various stages of fetal kidney development [2]. There are currently two main protocols for treating Wilms’ tumors: the protocol of the Children’s Oncology Group (COG), followed in North America, dictates primary surgery followed by chemotherapy [3], while the protocol of the Société International d’Oncologie Pédiatrique (SIOP) [4], followed in Europe and other countries, dictates preoperative chemotherapy followed by surgery and then postoperative chemotherapy. Both protocols recommend postoperative radiotherapy when there are indications for tumor spread or incomplete tumor removal.

Wilms tumors are pathologically classified according to their cell type composition: “Stromal” tumors contain a large fraction of cells that resemble the un-induced mesenchyme (nephrogenic zone stroma) of the developing fetal kidney, and sometimes additional tissues such as muscle and cartilage that, like the un-induced mesenchyme, also originate from the fetal mesoderm [2]. “Blastemal” tumors resemble the cap mesenchyme, which is a transient cellular compartment in the fetal developing kidney that contains the nephron progenitors. “Epithelial” tumors contain a significant fraction of poorly differentiated epithelial structures. However, in many cases the tumors contain a mixture of all three components and are classified as “mixed” or “triphasic”. The component proportions can vary significantly, resulting in a continuous range of tumor appearances [5].

In a previous study [6], we performed Pareto task inference on a dataset of microarray gene expression measurements from favorable histology Wilms’ tumors (FHWT’s). These samples were collected by the Children’s Oncology Group [7,8,9,10] from tumors that were surgically resected before chemotherapy—according to the COG protocol. We found that the tumors fill a triangle-shaped continuum in gene expression latent space, of which the vertices represent three idealized “archetypes”, and showed that these archetypes have predominantly renal blastemal, stromal, and epithelial characteristics that correlate well with the three major lineages of the developing fetal kidney. Moreover, we showed that advanced stage tumors tend to shift towards the blastemal archetype in latent space.

However, the heterogeneity of tumors that were already treated with chemotherapy before surgery—according to the SIOP protocol—might be quite different, since chemotherapy is known to alter the tumor’s histological features and distribution of subtypes [4]. We therefore set to characterize the transcriptional heterogeneity also in high-risk, blastemal type, post-operative chemotherapy Wilms’ tumors using a dataset of microarray gene expression measurements collected by Wegert et al. [11]. The tumors in this dataset were treated with chemotherapy according to the SIOP protocol but still contained a significant amount of remaining viable blastema after treatment, a situation that is known to be associated with worse prognosis. 

To characterize the continuous heterogeneity in this dataset of high-risk tumors, we used an unsupervised machine learning algorithm called grade of membership (GOM) or topic modeling, which is a form of “soft” clustering. As opposed to “hard” clustering algorithms, which seek to partition samples or genes into distinct subgroups [12,13], soft clustering algorithms allow each sample to have memberships in multiple clusters simultaneously. This method has been used for studying patterns of gene expression in tissues, for example, Dey et al. [14] demonstrated the use of grade of membership modeling in bulk gene expression data from 53 human tissues from the GTEx project. A similar approach has been used in single cell deconvolution algorithms [15,16] to estimate the proportions of different cell types in bulk gene expression profiles. In our study, we fit a topic model to a bulk gene expression dataset of high-risk, blastemal type, post-operative chemotherapy Wilms’ tumors, and show that each tumor can be modeled as a combination of three “idealized” tumor types or “topics”. This demonstrates the potential of topic models for characterizing continuous heterogeneity in cancer. 

## 2. Results

### 2.1. Pareto Task Inference Shows That Blastemal Type, Post-Operative Chemotherapy Wilms’ Tumors Fill a Triangle-Shaped Continuum in Latent Space That Is Bounded by Archetypes with Stromal, Epithelial, and Blastemal Characteristics

**Figure 1:**
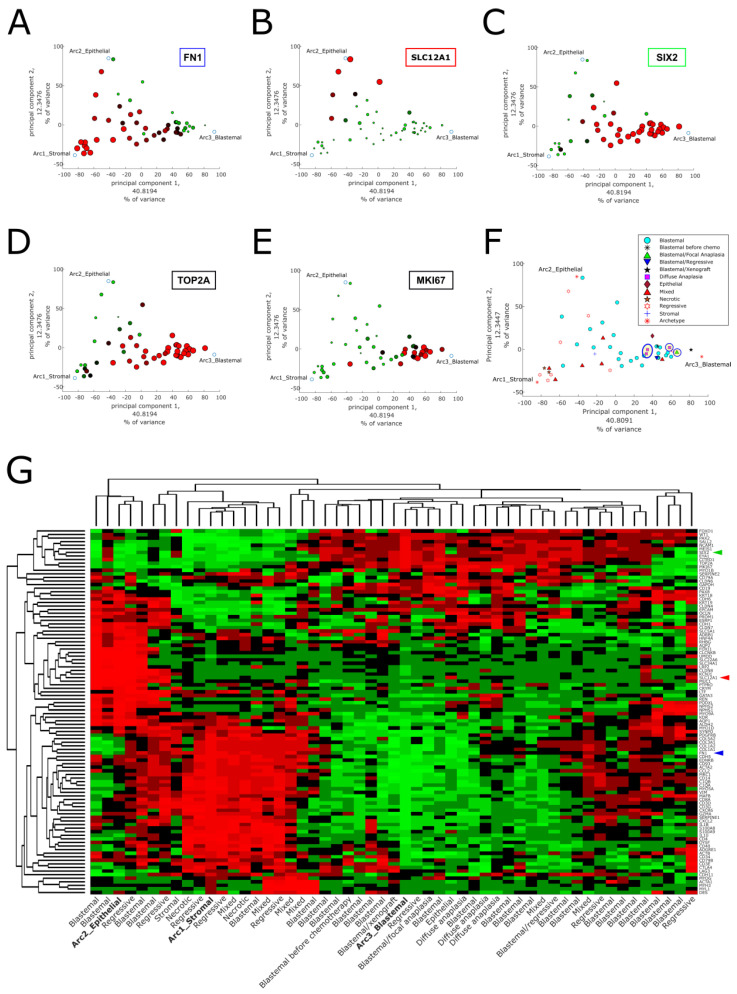
Pareto task inference shows that blastemal type, post-operative chemotherapy Wilms’ tumors fill a triangle shaped continuum in latent space that is bounded by archetypes with stromal, epithelial, and blastemal characteristics. (**A**–**E**) Shown are PCA plots of the tumors in our dataset along with the three calculated archetypes (the vertexes of the triangle). In order to identify the archetypes, in each panel we selected a gene marking one of the three main lineages of the fetal developing kidney and plotted the size and color of each point according to its expression level (red large—high expression, green small—low expression). It can be seen than *FN1*, a marker for the un-induced mesenchyme, is highly expressed towards the stromal archetype. Likewise, *SLC12A1*, which marks the renal epithelium, is highly expressed near the epithelial archetype, and *SIX2*, a marker for the cap mesenchyme, is predominantly expressed near the blastemal archetype. The genes *TOP2A* and *MKI67*, which mark cycling cells, are also highly expressed near the blastemal archetype. (**F**) A PCA plot of the tumors marked according to their reported histological type. It can be seen that tumors with anaplastic histology (diffuse or focal), which is considered least favorable, tend to cluster in the vicinity of the blastemal archetype. (**G**) A gene expression heatmap of 102 genes that are known from the literature to mark specific kidney lineages. The three arrowheads mark the genes from panels (**A**–**C**). It can be seen that the stromal, blastemal, and epithelial archetypes over-express genes that mark the un-induced mesenchyme, the cap mesenchyme, or renal epithelial tubular structures, respectively. Hierarchical clustering was done using the Matlab clustergram function with standardized rows (=genes), Euclidean distance metric, and average linkage.

We first downloaded a dataset containing gene expression microarray measurements from 53 high-risk, blastemal type, post-operative chemotherapy Wilms’ tumors that were published by Wegert et al. [11]. After preprocessing and data standardization, we performed principal components analysis (PCA) and found that the tumors form a triangle-shaped continuum in latent space, rather than discrete separated clusters (Figure 1A–F). Following the principle of Pareto task inference [17,18], we assume that the vertexes of this triangle-shaped geometric configuration represent “idealized” tumor components or cellular “archetypes” from which all the tumors within the triangle are composed. We therefore used the ParTI Matlab package developed by Alon and colleagues [17,19] to find these archetypes, which are the vertexes of the optimal fitting triangle encompassing the tumors in our dataset. The archetypes were then presented in the same space as the original data points (Figure 1A–F). 

In order to characterize and identify the three archetypes, we first chose a set of genes that mark the three main lineages in the developing kidney (the un-induced mesenchyme, the cap mesenchyme, and early renal epithelial structures) and examined their pattern of expression in latent space (Figure 1A–C). We found that *FN1*, a gene that is known to be highly expressed in the un-induced mesenchyme, is highly expressed near the first archetype. The membrane transporter gene *SLC12A1*, that is known to mark epithelial tubules in the developing kidney, is over expressed near the second archetype. The gene *SIX2*, which marks the cap mesenchyme, is highly expressed near the third archetype. We therefore labeled the three archetypes as “stromal”, “epithelial”, and “blastemal”, respectively. Moreover, we found that *TOP2A* and *MKI67*, genes that are known to be over-expressed in proliferating cells, are highly expressed near the blastemal archetype (Figure 1D,E), which is consistent with the proliferative and “aggressive” nature of blastemal tumors after chemotherapy [11]. 

To confirm our characterization of the three archetypes, we selected a set of 102 genes (Table S3) that are known from the literature to mark specific cell populations in the developing kidney. Then, we performed hierarchical clustering of the tumors in our dataset and their archetypes with respect to these genes (Figure 1G and Figures S1–S12). Indeed, it can be seen that markers for the un-induced mesenchyme (*COL1A1*, *COL3A1*, *COL5A2*, *FN1*, and *SERPINE1*) are over-expressed in the stromal archetype, markers of the cap mesenchyme (*SIX2*, *CITED1*, *EYA1*, and *SALL2*) are over-expressed in the blastemal archetype, and markers for the renal epithelial tubules (*SLC12A1*, *AQP2*, *LRP2*, and *UMOD*) are over-expressed in the epithelial archetype. We noticed that the stromal archetype also over-expresses genes characteristic to immune cells (*C1QA*, *C1QB*, *CCL2*, *CD14*, *CD93*, and *CXCL2*), which is consistent with the fact that the un-induced mesenchyme contains a relatively large number of infiltrating immune cells (e.g., macrophages). We also note that the stromal archetype over-expresses genes characteristic to muscle cells (*DES*, *MYL1*, *MYH3*, and *MYOG*), which is consistent with the fact that some stromal tumors have been known to contain muscle-like cells. 

**Figure 2:**
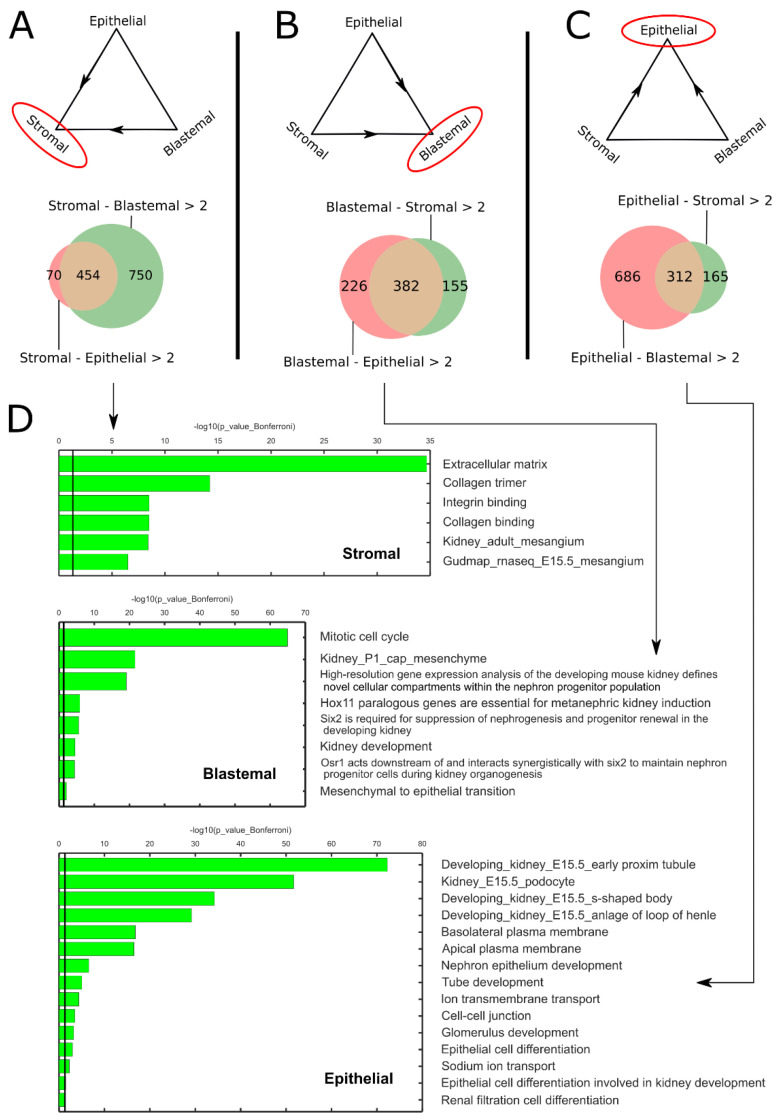
GO enrichment analysis shows that the stromal, blastemal, and epithelial archetypes are enriched for genes that are characteristic of the un-induced mesenchyme, the cap mesenchyme, and renal epithelium of the fetal developing kidney, respectively. (**A**–**C**) For each of the three archetypes, we selected a set of genes for which the log2-fold change was larger than two, with respect to the other two archetypes. These genes were used as input to Toppgene. (**D**) It can be seen that the stromal archetype over-expresses genes that are characteristic of the un-induced mesenchyme, for example, genes responsible for creating and maintaining the extracellular matrix. The blastemal archetype over-expresses genes that are characteristic of the cap-mesenchyme; for example, genes responsible for maintaining nephron progenitors and for the mesenchymal to epithelial transition (MET). Likewise, the epithelial archetype over-expresses genes that are characteristic of the fetal renal epithelium; for example, genes responsible for nephron epithelial differentiation and genes responsible for creation and maintenance of renal proximal tubules, S-shaped bodies, cell–cell junctions, and membrane transporters.

We further characterized the archetypes using GO enrichment analysis (Figure 2). For each of the three archetypes, we found a list of genes that are over-expressed (log2FC > 2) with respect to both of the other two archetypes (Figure 2A–C, Tables S4–S6). We then inserted the three lists of genes into Toppgene [20]. We found that the stromal archetype is enriched for genes typical to the un-induced mesenchyme, for example, components of the extracellular matrix (Figure 2D). The blastemal archetype is enriched for genes involved in maintenance of nephron progenitors and the mesenchymal to epithelial transition (MET), which are characteristic of the cap mesenchyme. Finally, genes enriched in the epithelial archetype are involved in cell–cell junctions, transport, and renal epithelial differentiation, which are typical to the early epithelial structures in the developing kidney. 

We also checked the relation between the reported tumor histology and clinical parameters to its location in latent space (Figure 1F and Figure S13–S23). We observed that tumors with anaplastic histology (diffuse or focal), which is considered least favorable [11], as well as tumors with mutations in the genes *SIX1*, *SIX2*, or *DROSHA*, tend to cluster in the vicinity of the blastemal archetype. This agrees with the higher incidence of *SIX1/2* mutations in tumors with chemotherapy-resistant blastema that was observed by Wegert et al. [11]. Likewise, we observed that the single blastema-only xenograft in the dataset is also located closest to the blastemal archetype more than any other tumor. This is consistent with previous observations that patient-derived xenografts significantly increase the percentage of their blastemal component from their first passage [21]. 

### 2.2. Topic Modeling Shows That Each Tumor Can Be Represented as a Unique Mixture of Three Hidden Topics with Blastemal, Stromal, and Epithelial Characteristics

**Figure 3:**
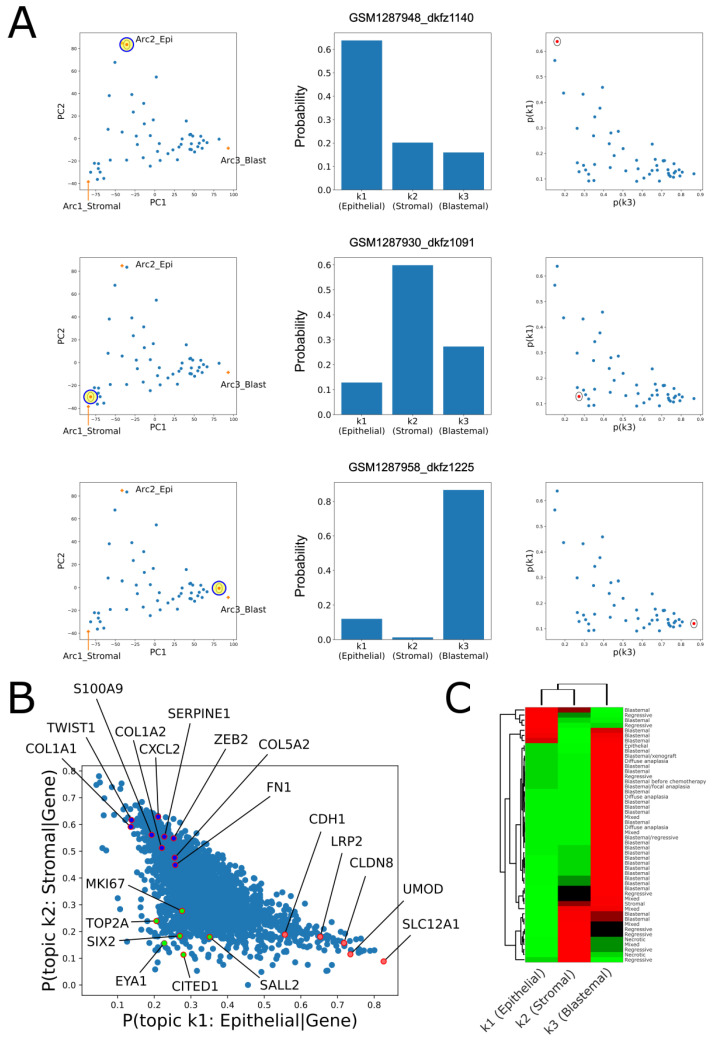
Topic modeling shows that each tumor can be represented as a unique mixture of three hidden topics with blastemal, stromal, and epithelial characteristics. (**A**) Shown are the fractions of topics (center and right panels) for selected tumors that are located near each of the three archetypes in latent space (left panels). The topic model predicts that each one of these tumors was generated primarily from a single topic. In particular, tumors near the epithelial topic contain a high fraction of topic no. 1 (top panels), tumors near the stromal topic contain a high fraction of topic no. 2 (middle panels), and tumors near the blastemal topic contain a high fraction of topic no. 3 (bottom panels). It can also be seen that the geometry of the topic simplex (right panels) resembles that of the PCA latent space (left panels). (**B**) The posterior probabilities of the three topics given the expression of specific genes show that the three topics have stromal, blastemal, and epithelial characteristics. The posterior probability of a topic given the expression of a specific gene represents the association between over-expression of that specific gene and the probability of that topic being represented in a tumor. It can be seen that over-expression of markers for the renal epithelial tubules (e.g., *CDH1*, *SLC12A1*, *LRP2*, and *UMOD*) is associated with high probability for the epithelial topic (topic no. 1), over-expression of markers for the un-induced mesenchyme (*COL1A1*, *COL1A2*, *COL5A2*, *FN1*, and *SERPINE1*) is associated with high probability for the stromal topic (topic no. 2), and over-expression of markers for the cap mesenchyme (*SIX2*, *CITED1*, *EYA1*, and *SALL2*) is associated with high probability for the blastemal topic (topic no. 3). (**C**) A heatmap of the topic composition of each tumor. It can be seen that, tumors with anaplastic histology (diffuse or focal), which is considered least favorable, as well as the single blastemal xenograft in the dataset, all contain a large fraction of the blastemal topic.

The fact that the tumors in our dataset create a triangle-shaped continuum in latent space suggests that each tumor can be represented as a unique mixture of three “idealized” tumor components. Therefore, in order to provide a more quantitative interpretation, we fitted a topic model with k = 3 hidden “topics” to our dataset [22] (see Materials and Methods). This allowed us to infer both the three latent topics (that presumably represent the “idealized” tumor components) and also the proportions of topics from which every single tumor is composed. We observed that, indeed, tumors located near each of the three vertexes of the triangle-shaped continuum are predominantly composed one out of the three topics (Figure 3A), whereas tumors located in-between the vertexes of the triangle or near its center are composed of multiple topics (Figures S24–S29 and Supplementary Data).

We next set out to identify the three topics (Figure S27). We observed that topic no. 1 over-expresses markers for the renal epithelial tubules (e.g., *CDH1*, *SLC12A1*, *LRP2*, and *UMOD*) and we therefore labeled it the “epithelial” topic. Likewise, topic no. 2 over-expressed markers for the un-induced mesenchyme (e.g., *COL1A1*, *COL1A2*, *TWIST1*, *ZEB2*, and *SERPINE1*) and we therefore labeled it the “stromal” topic, and topic no. 3 over-expressed markers for the cap mesenchyme (e.g., *SIX2*, *CITED1*, *EYA1*, and *SALL2*) and we therefore labeled it the “blastemal” topic.

We showed this also by calculating the posterior probabilities p(topic|gene) over all the genes for each of the three topics (Figure 3B). The posterior probability of a specific topic given the expression of a specific gene represents the association between expression of a transcript from that specific gene and the probability of that specific topic being represented in each of the tumors of our dataset. Indeed, we observed that that over-expression of markers for the renal epithelial tubules (e.g., *CDH1*, *SLC12A1*, *LRP2*, and *UMOD*) is associated with high probability for the epithelial topic (topic no. 1), over-expression of markers for the un-induced mesenchyme (*COL1A1*, *COL1A2*, *COL5A2*, *FN1*, and *SERPINE1*) is associated with high probability for the stromal topic (topic no. 2), and over-expression of markers for the cap mesenchyme (*SIX2*, *CITED1*, *EYA1*, and *SALL2*) is associated with high probability for the blastemal topic (topic no. 3). To further confirm this, we selected a list of genes characterizing each topic, that is, genes for which the posterior probability p(topic|gene)>0.5. Using GO enrichment analysis as before, we found that indeed, the three hidden topics over-express genes related to epithelial (resembling early renal tubular epithelium), stromal (un-induced mesenchyme-like), or blastemal (cap mesenchyme-like) cell types (Tables S7–S9).

We also checked the relation between the topic composition of each tumor to its histology and clinical parameters (Figure 3C and Figure S28). We found that tumors with anaplastic histology (diffuse or focal), which is considered least favorable, as well as the single blastemal xenograft in the dataset, and also tumors with mutations in the genes *SIX1*, *SIX2*, or *DROSHA*, all contain a larger fraction of the blastemal topic.

### 2.3. Cellular Deconvolution Indicates That Each Tumor Is Composed of a Unique Mixture of Cell Populations Resembling Those of the Fetal Kidney

**Figure 4:**
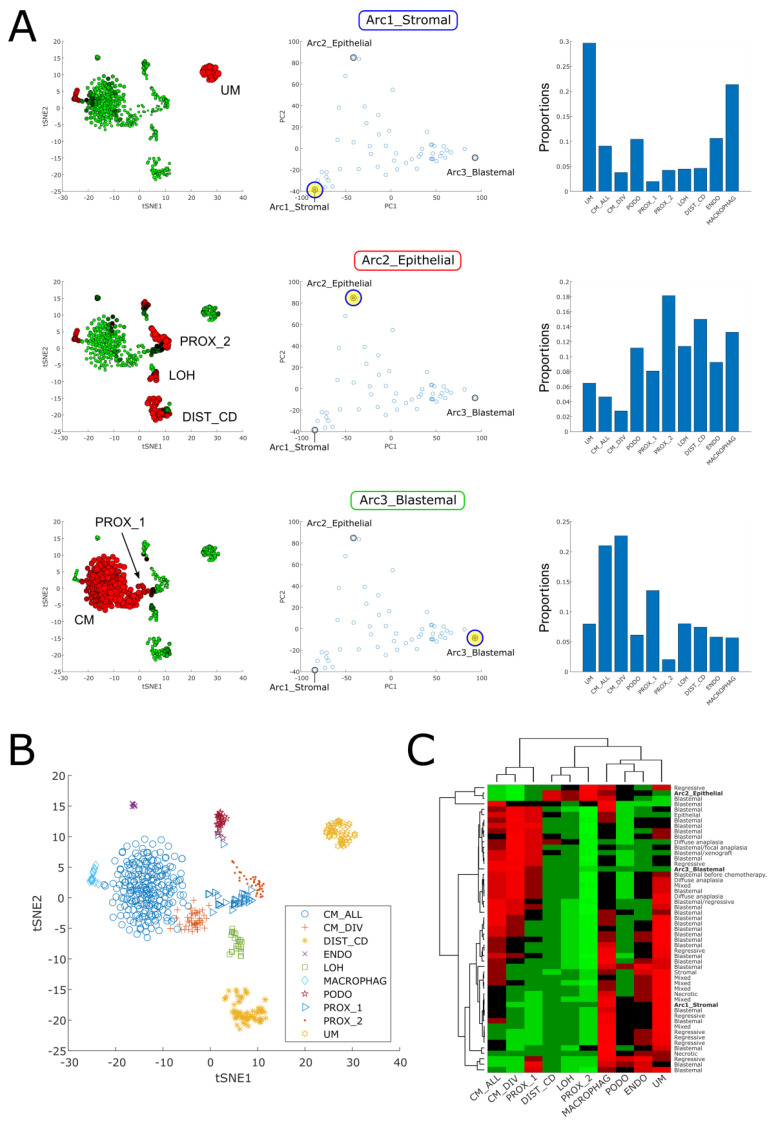
Cellular deconvolution indicates that each tumor is composed of a unique mixture of cell populations resembling those of the fetal kidney. (**A**) Shown is a cellular deconvolution of the stromal, blastemal, and epithelial archetypes. The left panels show tSNE plots of the reference single cell RNAseq dataset from the developing mouse fetal kidney that was used for the deconvolution. The size and color of each point (=single cell) corresponds to the proportion of that cell in the specific de-convolved tumor (red large—high expression, green small—low expression). The middle panels highlight the relevant tumor or archetype in the PCA latent space. The right panels show bar plots of the predicted proportions of each of the 10 cell types in the reference single-cell matrix. It can be seen that the stromal archetype is composed primarily from cells resembling the un-induced mesenchyme (UM) and macrophages, the epithelial archetype is composed mainly of epithelial tubular cells resembling those of the fetal proximal tubule (PROX_2), the Loop of Henle (LOH), and the distal tubule (DIST_CD), and the blastemal archetype is composed primarily from cells resembling the cap mesenchyme (CM), along with some very early epithelial tubular structures (PROX_1). (**B**) The different cell populations marked on a tSNE plot of the reference single cell RNAseq dataset from the developing mouse fetal kidney that was used for cellular deconvolution (CM—cap mesenchyme, DIST_CD—distal tubule and collecting duct, ENDO—endothelial, LOH—loop of Henle, MACROPHAG—macrophages, PODO—podocytes, PROX_1—early epithelial structures such as C/S-shaped bodies, PROX_2—proximal tubule, UM—un-induced mesenchyme). (**C**) A heatmap of the cell type proportions from which each tumor is composed, as predicted by cellular deconvolution. It can be seen that most of the tumors with reported blastemal histology contain a significant proportion of cells resembling those of the cap mesenchyme (CM), as expected. Likewise, tumors with anaplastic histology (diffuse or focal), as well as the single blastemal xenograft in our dataset, also contain a significant fraction of cells resembling the cycling cap mesenchyme cells (CM_DIV).

We next used cellular deconvolution [16] to infer a more detailed cellular composition of each tumor. Since cells in Wilms’ tumors closely resemble those of the fetal kidney, we used a previously published single-cell gene expression dataset from a mouse fetal developing kidney [23] as reference. The cellular deconvolution algorithm was used to predict the proportions of ten cell types from the developing kidney within each of the tumors and archetypes (Figure 4 and Figures S30–S32).

We observed that the stromal archetype is composed primarily of cells resembling the un-induced mesenchyme (UM) and macrophages, the epithelial archetype is composed mainly of epithelial cells resembling those of the fetal proximal tubule (PROX_2), the Loop of Henle (LOH), and the distal tubule (DIST_CD), and the blastemal archetype is composed primarily of cells resembling the cap mesenchyme (CM), along with some cells resembling very early epithelial tubular structures (PROX_1). Tumors that are near the archetypes in latent space are likewise composed primarily from these cell types, while tumors in between the archetypes or in the middle of the triangle shaped continuum are composed of more heterogeneous mixtures of the different cell populations (Figure S31 and Supplementary Data).

We also correlated the reported histology with the cell type repertoire inferred by cellular deconvolution (Figure 4C and Figure S32). We found that most of the tumors with reported blastemal histology contain a significant proportion of cells resembling those of the cap mesenchyme (CM_ALL), as expected. Likewise, tumors with anaplastic histology (diffuse or focal), as well as the single blastemal xenograft in our dataset, and also tumors reported to contain mutations in the genes *SIX1*, *SIX2*, or *DROSHA*, contained a significant fraction of cells resembling the cycling cap mesenchyme cells (CM_DIV) in the fetal kidney. This is in agreement with the findings of Wegert et al. [11] that blastemal-type Wilms tumors with mutations in *SIX1* or *SIX2* have a gene expression signature of proliferation and kidney progenitors.

## 3. Discussion

In this study, we found that high-risk blastemal-type Wilms’ tumors, that is, tumors that were treated with chemotherapy according to the SIOP protocol but still contained a significant amount of remaining viable blastema after treatment, form a triangle shaped continuum in the latent space spanned by the first two principal components. Using Pareto task inference and GO enrichment analysis, we showed that the vertices of this triangle represent “stromal”, “blastemal”, and “epithelial” cellular archetypes that correspond to the three main lineages in the developing fetal kidney—the un-induced mesenchyme, the cap mesenchyme, and the renal tubular epithelium. We then used topic modeling to fit a generative probabilistic model to our dataset and showed that each tumor can be represented as a unique mixture of three hidden “topics” with blastemal, stromal, and epithelial characteristics. We complemented this by performing cellular deconvolution with respect to an independently measured single-cell gene expression dataset from a fetal kidney in order to represent each tumor along the continuum as a unique combination of fetal kidney-like cell states. All three approaches yielded consistent results, thus further highlighting the relationship between Wilms’ tumors and kidney development.

We observe similar triangle-shaped formations in latent space and similar archetypes in both favorable histology Wilms’ tumors (FHWT’s) [6], that were surgically resected before chemotherapy according to the COG protocol, as well as in high-risk blastemal type Wilms’ tumors, that were treated with chemotherapy according to the SIOP protocol. This indicates that the triangle-shaped continuum formed by Wilms’ tumors in latent space is a conserved and intrinsic property of Wilms’ tumor heterogeneity. Note that in high-risk blastemal type Wilms’ tumors we were not able to observe a shift of advanced-stage tumors towards the blastemal archetype, as we observed in favorable histology Wilms’ tumors. This indicates that chemotherapy alters the proportions of cell populations in each tumor such that the dominant predictor for clinical outcome is the presence or absence of a blastemal component, whereas other features become relatively less important. Another possibility is that the number of samples in the present study is too small to observe this phenomenon. We also note that integration of both datasets is difficult since the studies were performed with microarrays of different types and there is a large technical bias between them. We believe that this problem will be mitigated in the future as more and more tumors are analyzed using RNA sequencing at the bulk and single-cell levels.

## 4. Materials and Methods

### 4.1. Gene Expression Datasets 

A total of 53 CEL files were downloaded from the GEO database (accession number GSE53224). We also received a table connecting the microarray ID’s from the GEO database (GSM1287918_dkfz1079, GSM1287919_dkfz1080, …) to the tumor identifiers from Table S2 in the original publication by Wegert et al. [11] (WT055, WT056, …), from Prof. Manfred Gessler, who is one of the authors of the original study (see data and metadata in Tables S1 and S2). 

### 4.2. Data Preprocessing

Microarray data preprocessing was performed with the “affy” R package using the “rma” function with default parameters. We created a gene expression table by choosing, for each gene, the probe-set with maximal mean value across all arrays using the “collapseRows” R function from the WGCNA package.

After performing PCA, three of the samples (GSM1287965, GSM1287967, and GSM1287968) were observed to be clear outliers in the latent space formed by the first three principal components. Since the heterogeneity that these tumors represent cannot be effectively modeled with only three samples, combined with the fact that the algorithms we employed might be susceptible to these outliers, we decided to remove these samples from the rest of the analysis. 

### 4.3. Data Visualization and Clustering

PCA was performed in Matlab using the “pca” function with the default SVD algorithm and centered features. For hierarchical clustering we used the Matlab function “clustergram” with standardized rows (=genes or features), Euclidean distance metric, and average linkage, except when otherwise specified. 

### 4.4. Archetype Analysis

The archetypes and best fitting simplex containing the data points (=tumors) were calculated using the “ParTI_lite” matlab function (https://www.weizmann.ac.il/mcb/UriAlon/download/ParTI, accessed on 15 January 2022). Pareto Task Inference (ParTI), is a method for inferring biological tasks from high dimensional data [17]. The function finds the shape of the best fitting polytope (triangle, tetrahedron, etc.) which encompasses the data points. The vertices of this polytope, or “archetypes”, represent biological tasks, and data points specialize at each task according to their distance from the archetypes. The identity of the tasks can be inferred from features enriched near the archetypes. We first log-transformed the expression data and used the default Sisal [24] algorithm to find the best fitting simplex.

For Gene Ontology Enrichment analysis, lists of genes found to be over-expressed in each of the archetypes were used as inputs to ToppGene [20]. Venn diagrams were prepared using the matplotlib function “Venn2” in Python with default parameters.

### 4.5. Topic Modeling

“Topic models” or “grade of membership models” are used in natural language processing to model documents that contain words from different “topics” [25]. Given a set of documents and an assumed number of topics, k, from which they are composed, it is possible to infer the best fitting parameters of the model by using the Expectation Maximization (EM) algorithm, and thus discover both the k latent topics as well as their proportions in each individual document. Other applications are in population genetics to model individuals with mixed ancestry, and in gene expression datasets to model samples with partial memberships in multiple biologically-distinct clusters [14].

A type of topic model called the Latent Dirichlet Allocation (LDA) model assumes that the given set of documents can be characterized by a Dirichlet distribution with concentration parameters $\alpha_{1},\alpha_{2},\ldots ,\alpha_{k}$. Each individual document in the set is generated by mixing k latent topics with proportions described by the random numbers $\theta_{1},\theta_{2},\ldots ,\theta_{k}$ (whose sum equals to one) that are chosen from the Dirichlet distribution. Each word in the document is independently generated by first selecting one of these topics (according to the probabilities $\theta_{1},\theta_{2},\ldots ,\theta_{k}$) and then sampling a word from the dictionary (that is, the distribution over words) associated with the chosen topic. 

In our case, each document corresponds to a tumor, each topic corresponds to an “idealized” tumor, each word in the vocabulary (“word bag”) corresponds to a gene, and the number of occurrences of a specific word in given document (or topic) corresponds to the number of mRNA molecules, or the expression level, of that specific gene in the specific tumor (or “idealized” tumor). Thus, fitting a topic model to a dataset of gene expression profiles from a set of tumors enables us to infer the latent topics (that presumably represent “idealized” tumors) as well as the proportions of topics from which every single tumor is composed.

In this study, the parameters of the topic model were learned using the “fit_topic_model” function from the R package “fastTopics” [22] (version 0.4-11). The number of latent topics was set between k = 2, 3, …, 10. The “fastTopics” package estimates the parameters of the topic model by using non-negative matrix factorization (NMF) based on maximizing a Poisson log-likelihood function. Using default settings, the “fit_topic_model” function performs 100 iterations of the expectation maximization (EM) algorithm followed by 100 iterations of the coordinate descent (CD) algorithm. The outputs of the learning algorithm are: (1) The “Loadings” L matrix which contains the proportions of the k topics $\theta_{1},\theta_{2},\ldots ,\theta_{k}$ in each tumor; (2) The “Factors” F matrix which contains the probability $p\left(gene|topic\right)$ of a transcript from a given gene being expressed in each one of the k topics. Log-fold changes between topics were computed using the “fastTopics” function “de_analysis”. 

One way to visualize the association between a specific gene and each of the k topics is to calculate the “posterior probabilities” $p\left(topic|gene\right)$ for each topic and compare them. The term $p\left(topic|gene\right)$ is the probability of a specific topic being represented (in any sample of our dataset) given the expression of a single transcript from that specific gene. To calculate the posterior probabilities, we used Bayes’ theorem:

$$p\left(topic|gene\right)=\frac{p(gene|topic)\cdot p\left(topic\right)}{\sum_{topic}p(gene|topic)\cdot p\left(topic\right)}$$

where, for simplicity, we set the prior $p\left(topic\right)=1/3$ for each topic. Using this method, we were able to find sets of genes that are most associated with each of the k = 3 inferred topics and deduce their biological identity.

Choosing k, the number of topics for topic modeling, is often a “thorny” issue. In general, there is no one “true” value for k, and sometimes different values can even complement each other [14]. In our case, we chose k = 3 topics since the tumors form a triangle in latent space which indicates that they can be modeled as mixtures over three topics.

### 4.6. Cellular Deconvolution

Single-cell deconvolution algorithms estimate the proportion of different cell types present in a heterogeneous mixture bulk sample. In this study we used the Cell Population Mapping (CPM) algorithm [16] which uses both a reference single-cell expression matrix and the “cell-state space” of this reference matrix. The “cell-state space” contains the coordinates of each single-cell in latent space [26] and is obtained using algorithms such as PCA, tSNE, or UMAP. This latent space is used by the CPM algorithm to visualize cell trajectories and cell type information inside each bulk sample.

CPM consists of two steps: a deconvolution, or inference, step, and an extrapolation step. In the deconvolution step, the unknown abundance of each reference cell in the bulk expression sample is inferred using linear support vector regression (SVR). This step is repeated N different times, where each time a randomly chosen subset (of size “modelSize”, default value = 50) of the reference single cell matrix is used for the regression. The number of repetitions, N, is chosen such that each cell will be sampled at least a pre-selected number of times (“minSelection”, default value = 5). At the end of the deconvolution step, the predicted abundance values of each single cell in the bulk sample are obtained by averaging over the N runs. In the extrapolation step, CPM infers the abundance of each candidate cell-state in the bulk sample. This is done by averaging the cell abundance values inferred earlier over each cell’s Nd (“neighborhoodSize”, default size = 10) nearest neighbors in the reference cell-state space, thus creating a cell abundance that is “smoothed” over the reference cell-state space.

In this study, we used the “scbio” R package which accompanies the CPM paper (https://github.com/amitfrish/scBio, accessed on 28 January 2022), using a reference single-cell dataset from the mouse fetal kidney [23] and this dataset’s tSNE embedding for the reference cell-state space. We used scbio’s default values for the modelSize, minSelection, and neighborhoodSize parameters. We also set the parameter “quantifyTypes” = T to quantify the proportions of the different cell types in each bulk matrix in addition to the abundance values of each reference single-cell. 

## Data Availability

A total of 53 CEL files were downloaded from the GEO database (accession number GSE53224). We also received a table connecting the microarray ID’s from the GEO database (GSM1287918_dkfz1079, GSM1287919_dkfz1080, …) to the tumor identifiers from Table S2 in the original publication by Wegert et al. [11] (WT055, WT056, …), from Prof. Manfred Gessler, who is one of the authors of the original study (see Tables S1 and S2).

## Supplementary Materials

The following supporting information can be downloaded at: https://www.mdpi.com/article/10.3390/ijms24043532/s1.
