# Supplementary material for: Characterization of Continuous Transcriptional Heterogeneity in High-Risk Blastemal-Type Wilms’ Tumors Using Unsupervised Machine Learning
Source: Int J Mol Sci. 2023 Feb 9;24(4):3532. doi: 10.3390/ijms24043532 (PMC9965420; doi:10.3390/ijms24043532)
Supplement: Supplementary file 1 [file ijms-24-03532-s001.zip › ijms-2132028-supplementary figures.pdf]

Supplementary Information for: Characterization of Continuous Transcriptional Heterogeneity in High-Risk Blastemal-Type Wilms' Tumors Using Unsupervised Machine Learning

Yaron Trink [1], Achia Urbach [2], Benjamin Dekel [3], Peter Hohenstein [4], Jacob Goldberger [1] and Tomer Kalisky [1,*]

[1] Faculty of Engineering and Bar-Ilan Institute of Nanotechnology and Advanced Materials (BINA), Bar-Ilan University, Ramat Gan 5290002, Israel
[2] The Mina and Everard Goodman Faculty of Life Sciences, Bar-Ilan University, Ramat Gan 5290002, Israel
[3] Pediatric Stem Cell Research Institute and Division of Pediatric Nephrology, Edmond and Lily Safra Children's Hospital, Sheba Medical Center, Tel-Hashomer 5262000, Israel
[4] Department of Human Genetics, Leiden University Medical Center, 2300 RC Leiden, The Netherlands
* Correspondence: tomer.kalisky@biu.ac.il; Tel.: +972-3-738-4656

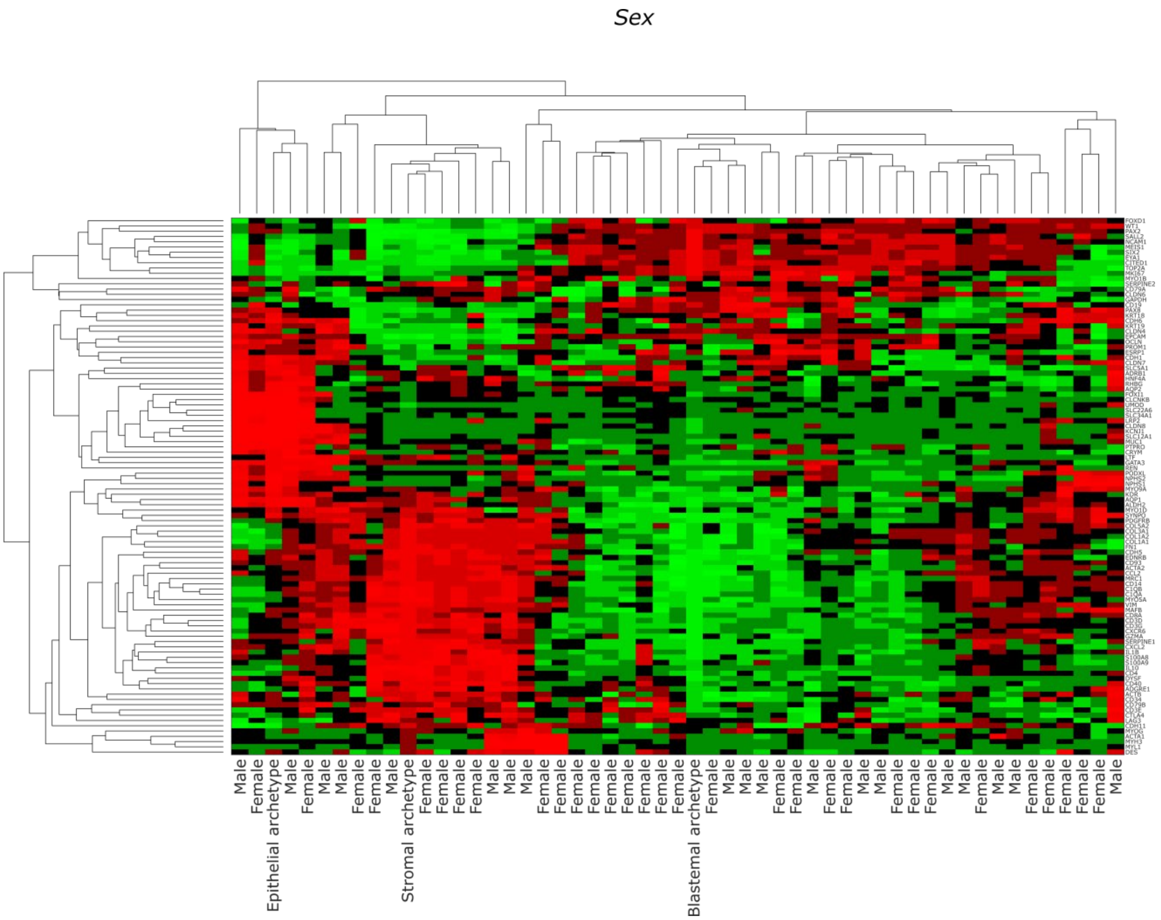

Figure S1: A heatmap of selected genes vs. tumors and archetypes showing reported **patient sex** (male/female).

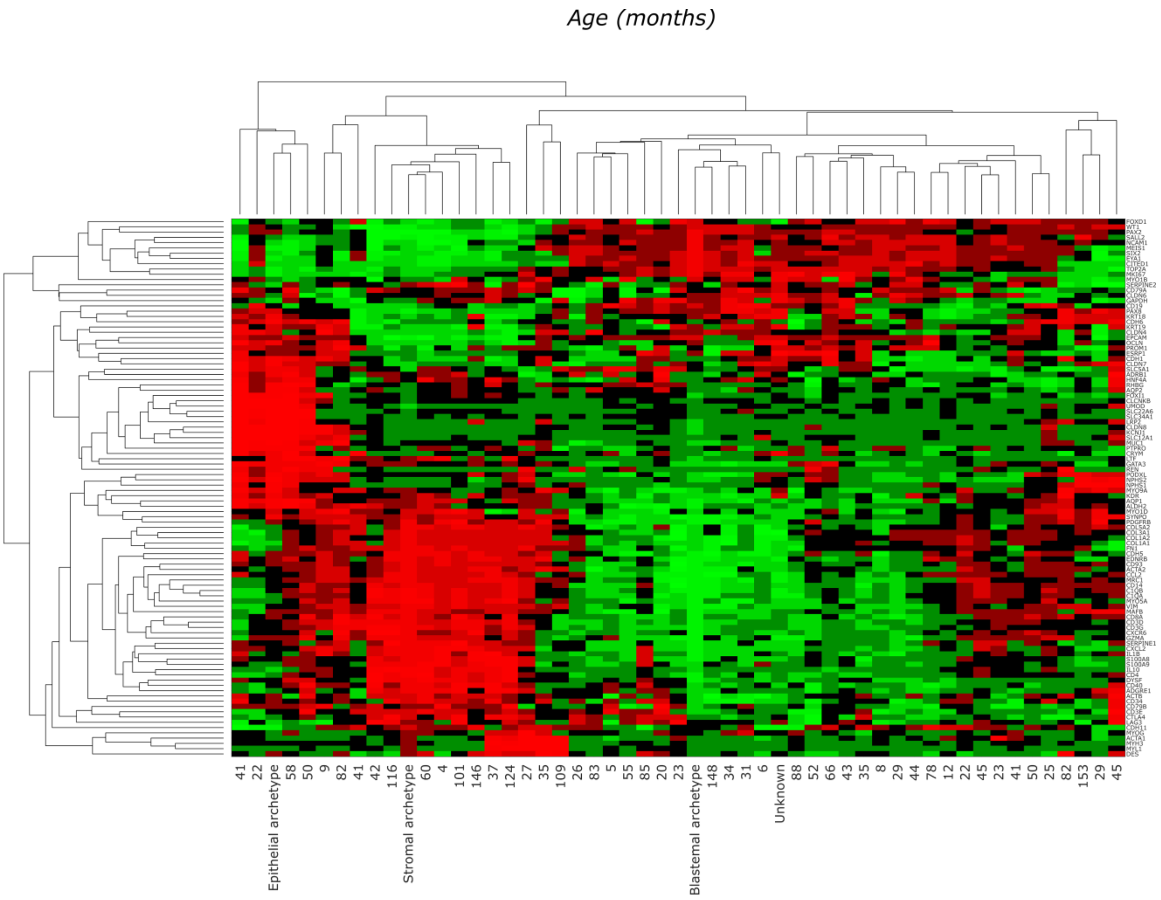

Figure S2: A heatmap of selected genes vs. tumors and archetypes showing **patient age** (in months) at diagnosis.

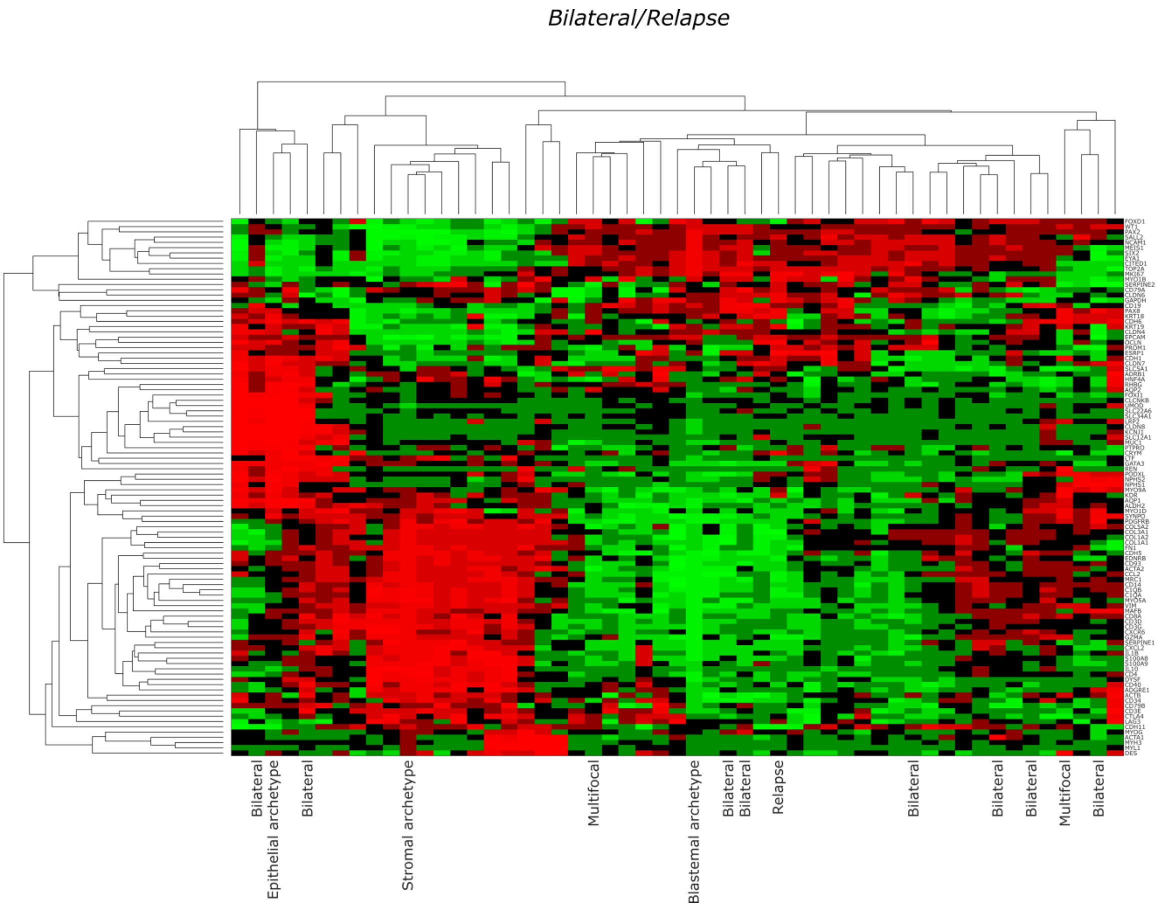

Figure S3: A heatmap of selected genes vs. tumors and archetypes showing clinical data (**bilateral and/or relapse**).

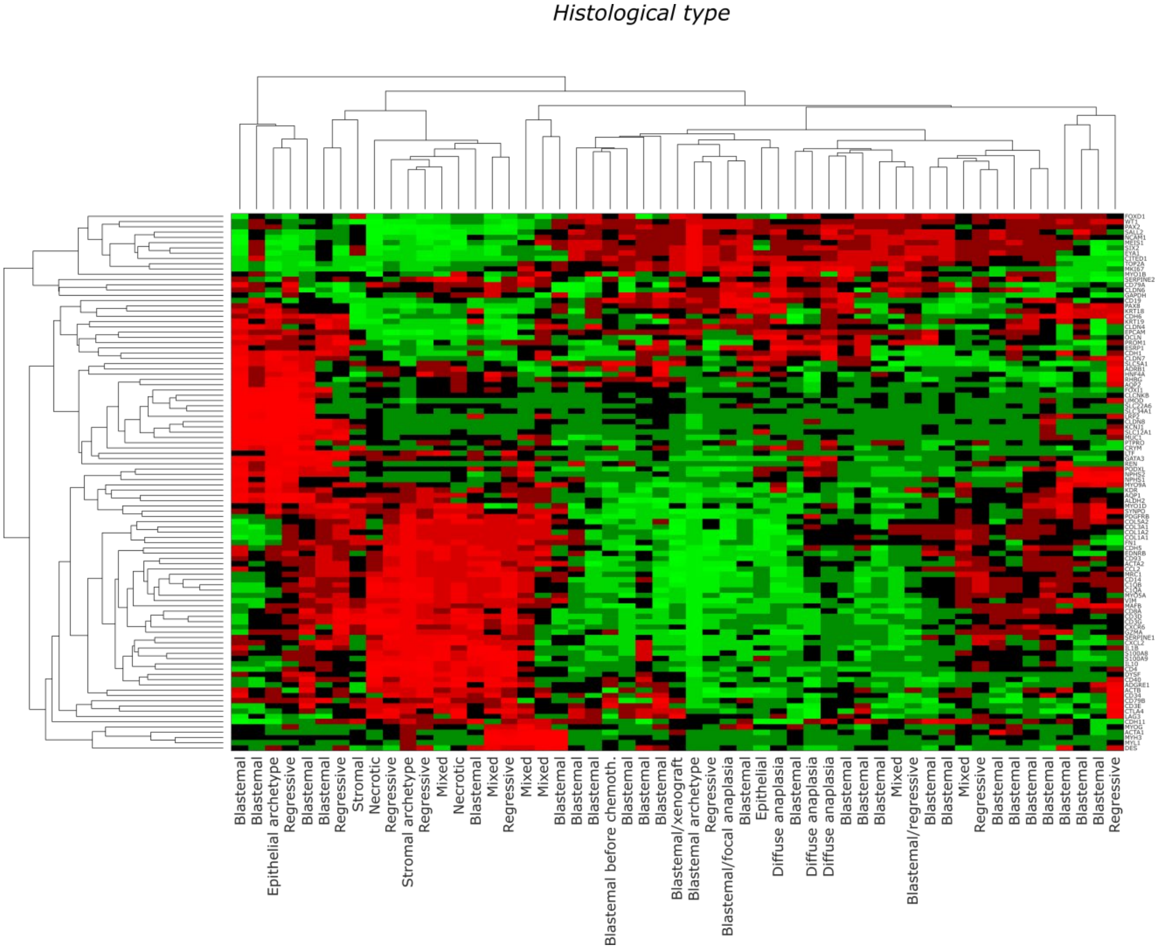

Figure S4: A heatmap of selected genes vs. tumors and archetypes showing the histological type.

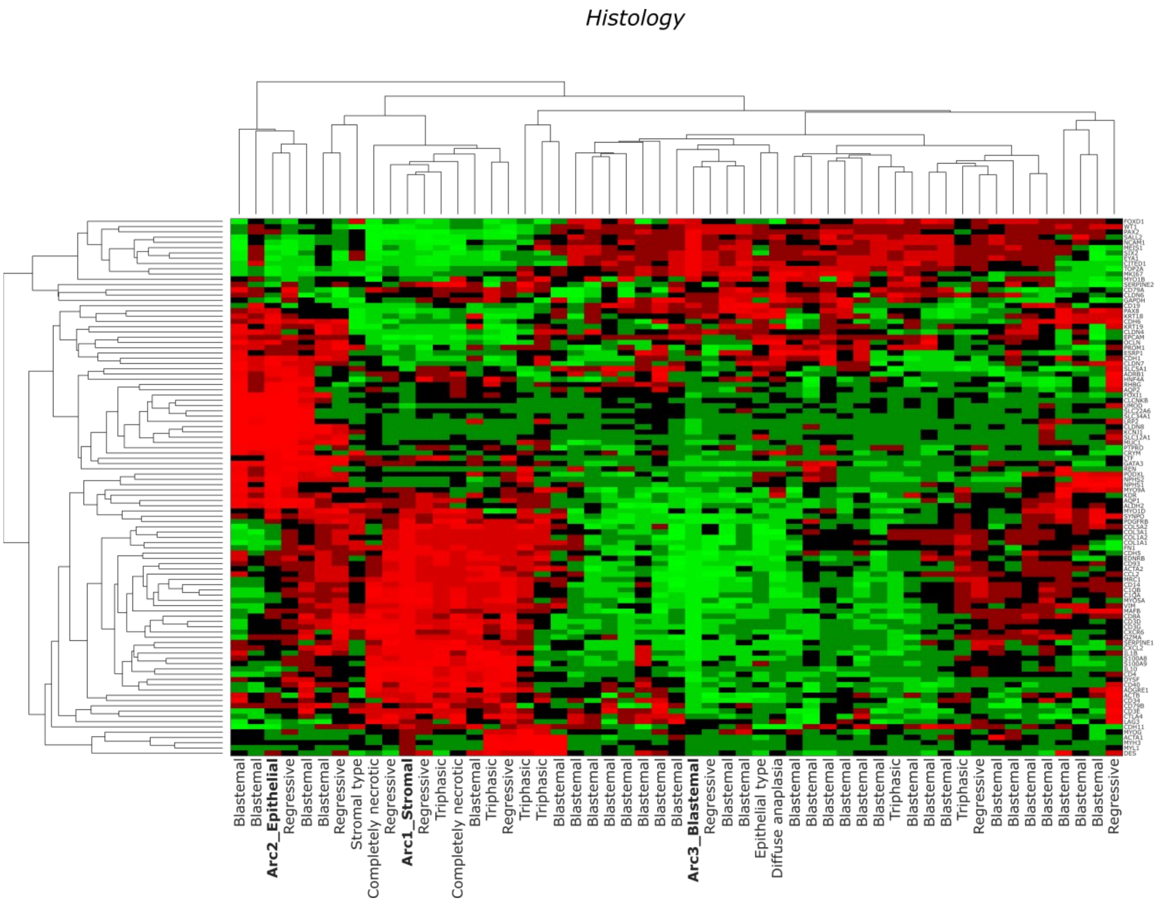

Figure S5: A heatmap of selected genes vs. tumors and archetypes showing tumor histology.

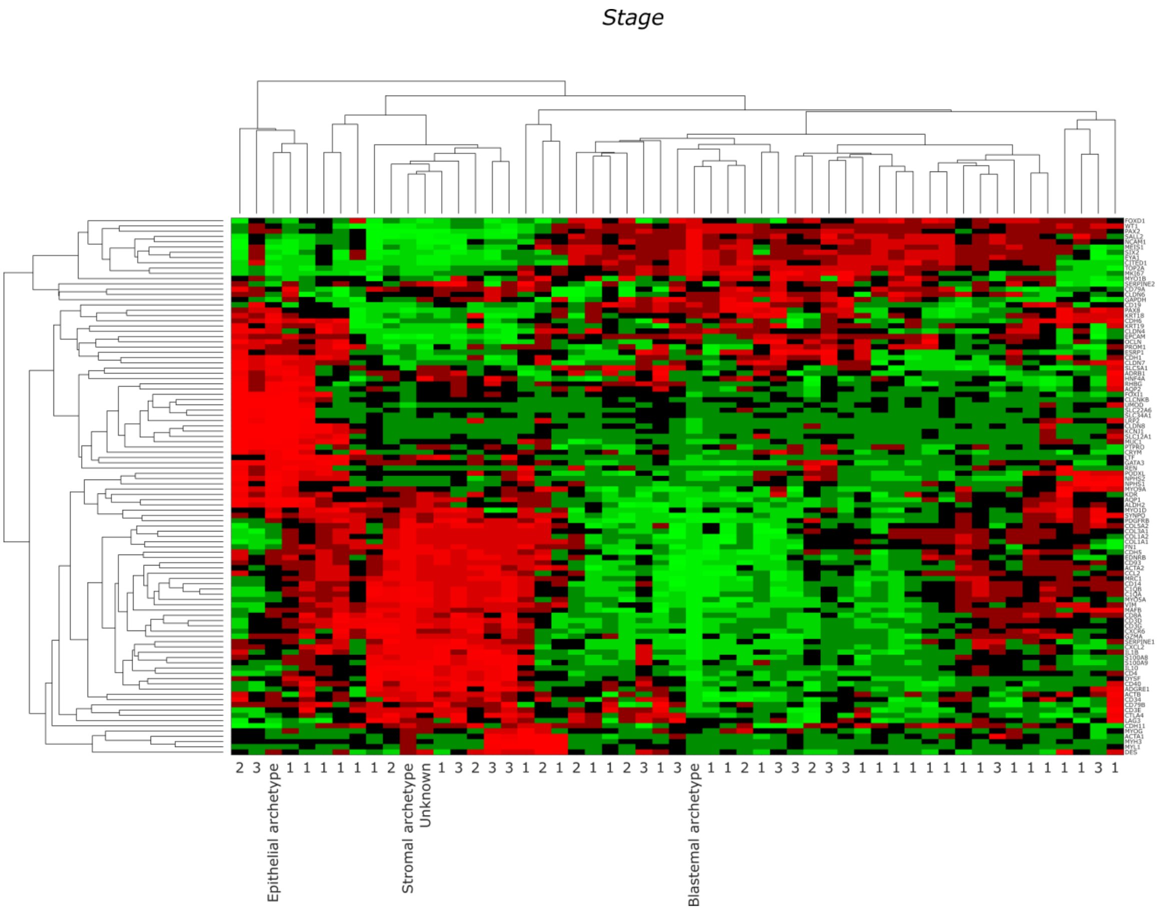

Figure S6: A heatmap of selected genes vs. tumors and archetypes showing reported **tumor stage** at diagnosis.

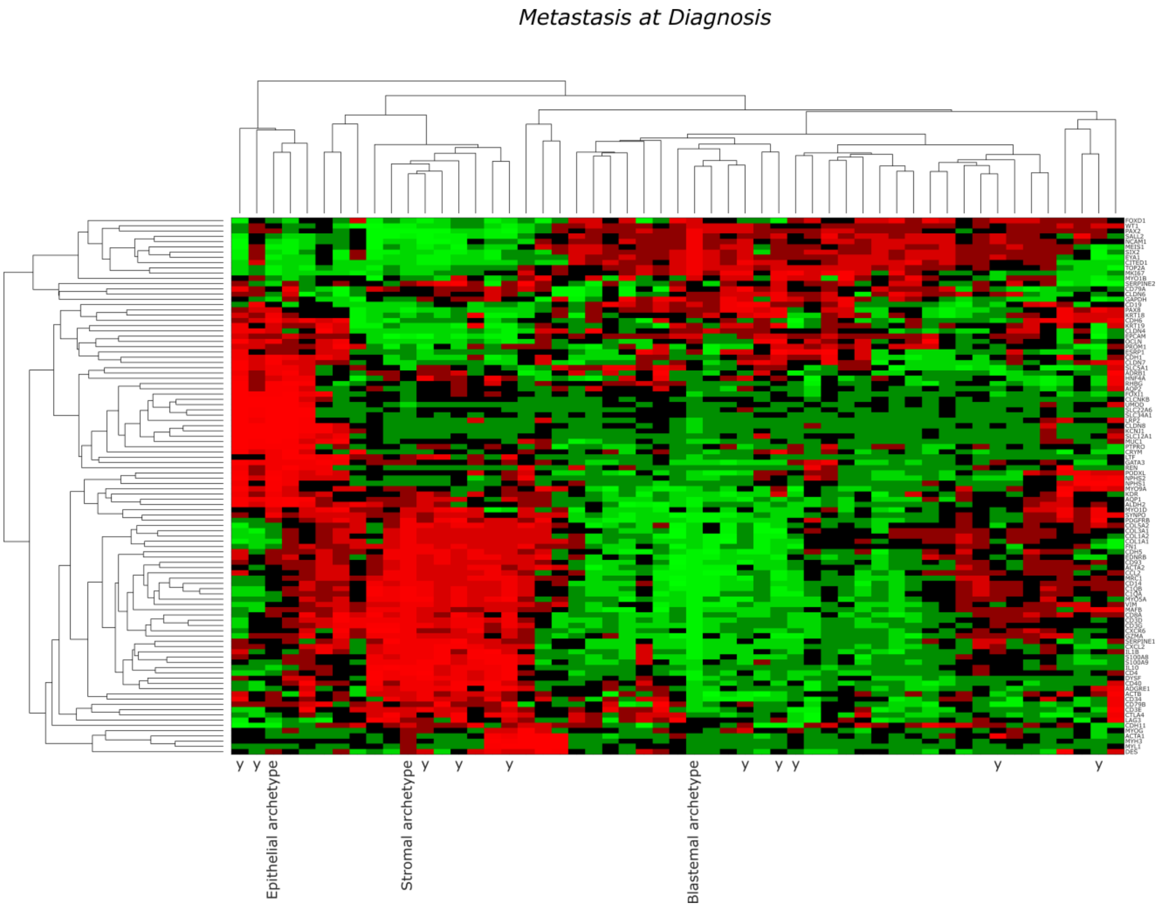

Figure S7: A heatmap of selected genes vs. tumors and archetypes showing reported **metastasis at diagnosis**.

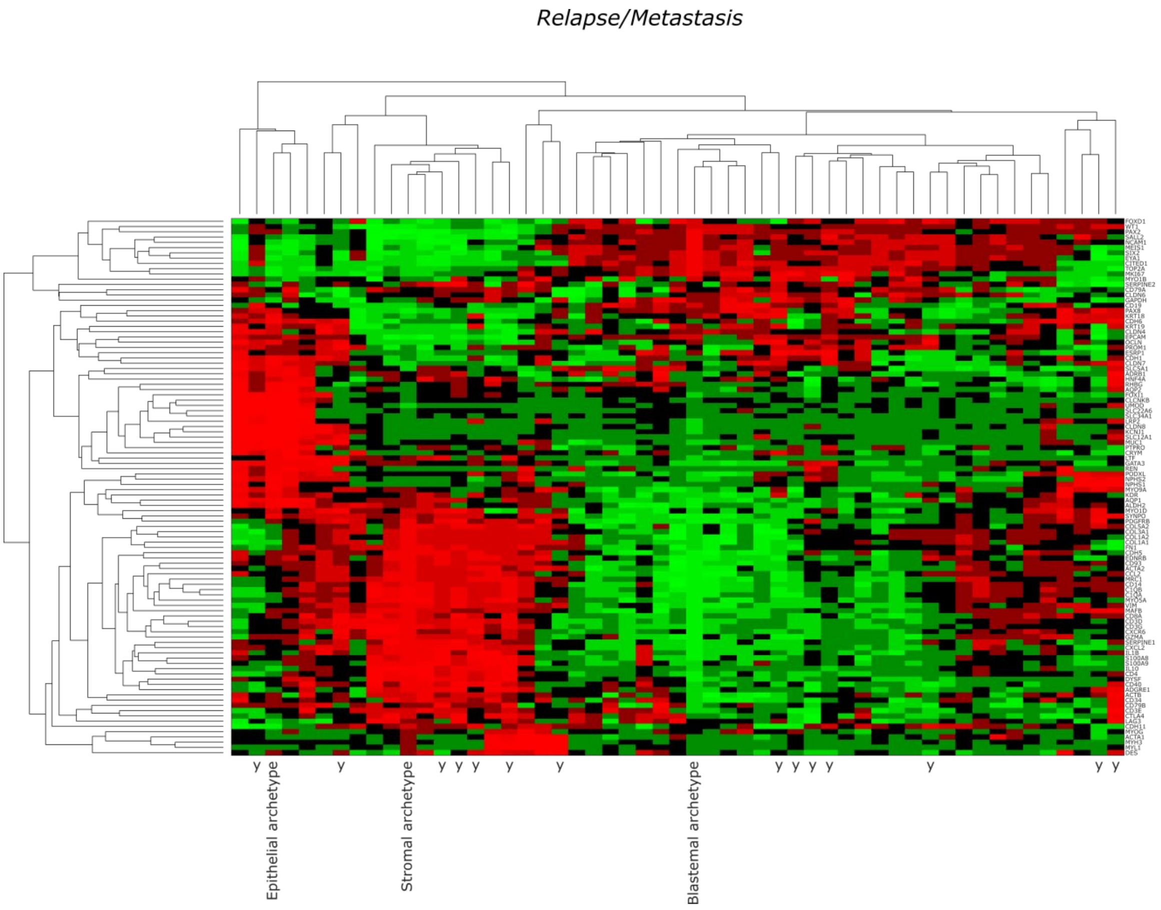

Figure S8: A heatmap of selected genes vs. tumors and archetypes showing the reported status of the tumor (**relapse/metastasis**).

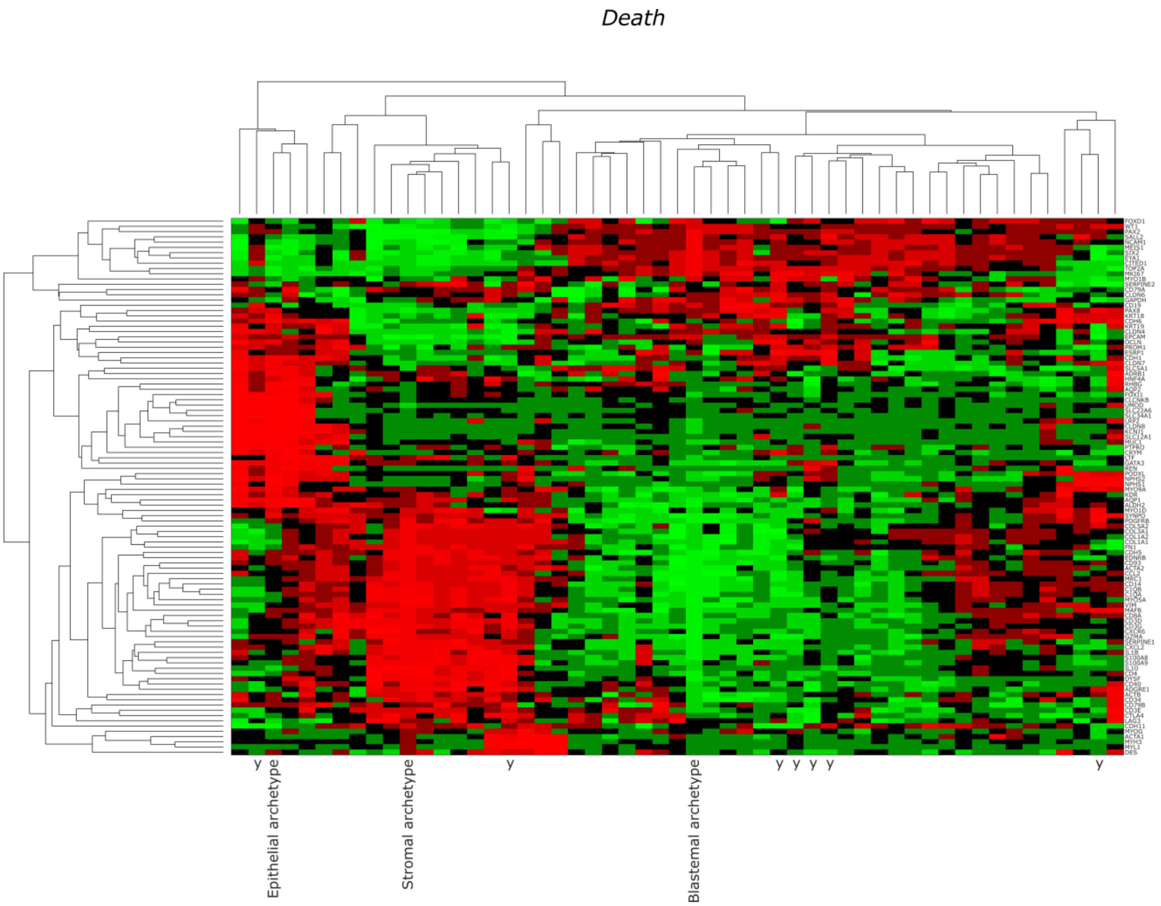

Figure S9: A heatmap of selected genes vs. tumors and archetypes showing the reported status of the patient (**death**).

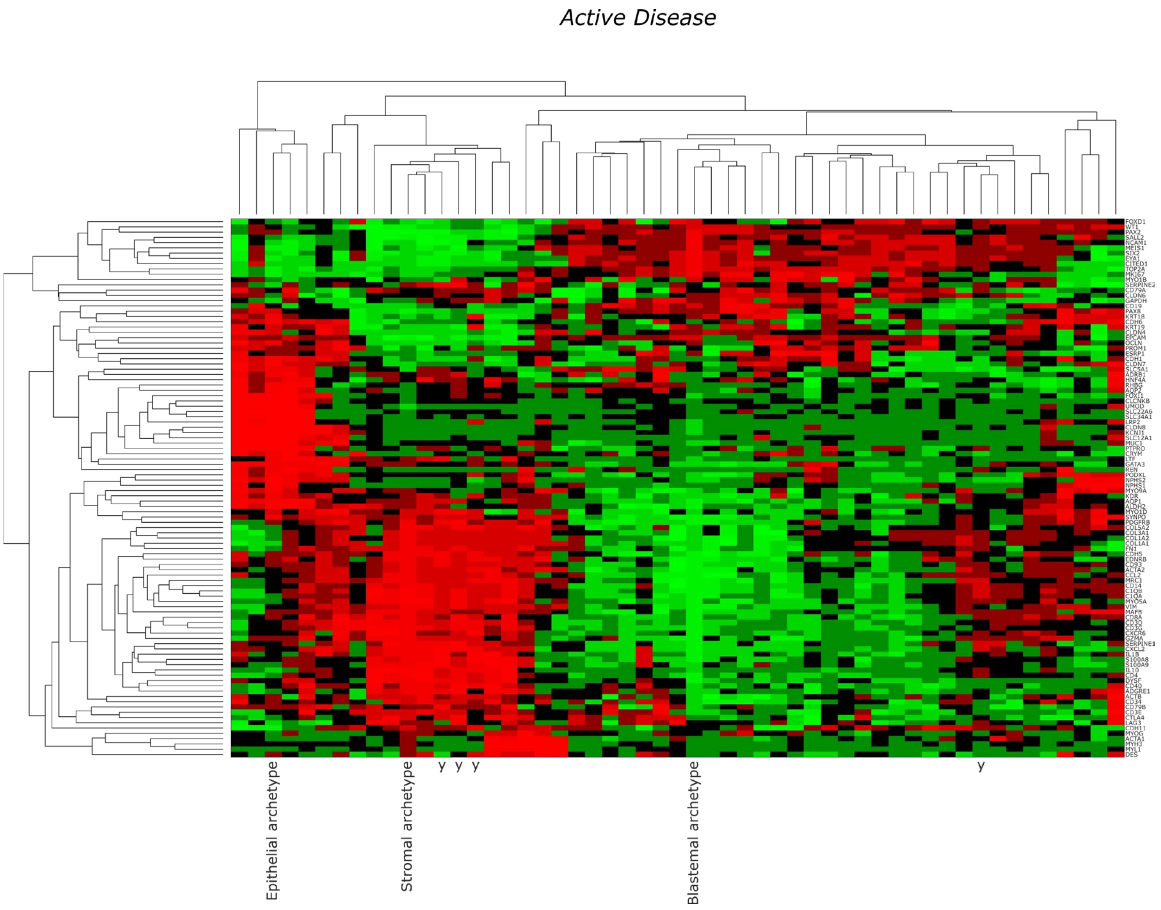

Figure S10: A heatmap of selected genes vs. tumors and archetypes showing the reported activity of the tumor (**active disease**). It can be seen that most tumors from patients with active disease in this dataset tend to cluster near the stromal archetype.

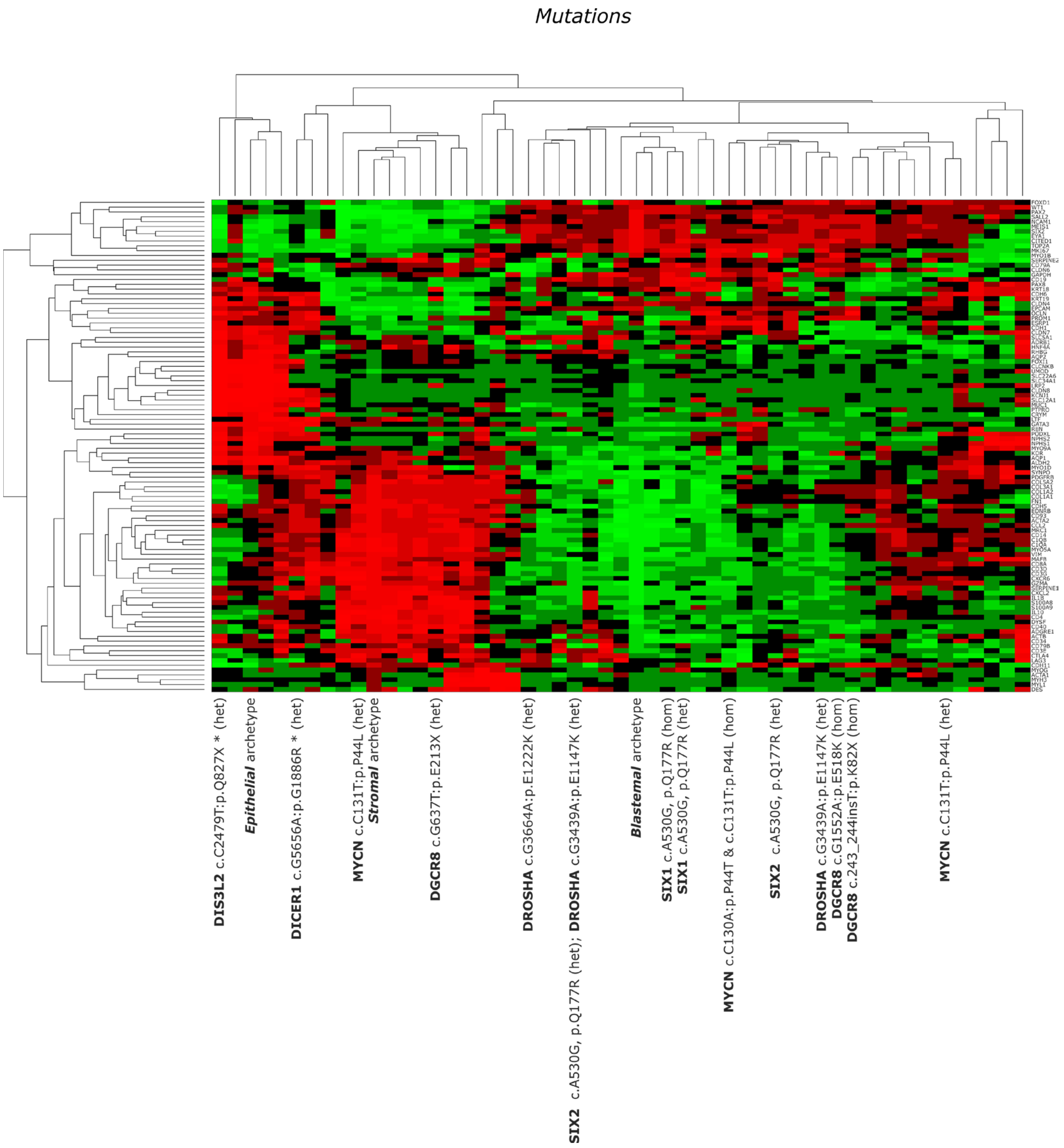

Figure S11: A heatmap of selected genes vs. tumors and archetypes showing reported **mutations**. It can be seen that mutations in the genes SIX1, SIX2, or DROSHA tend to cluster with the more blastemal tumors.

Heatmap DKFZ

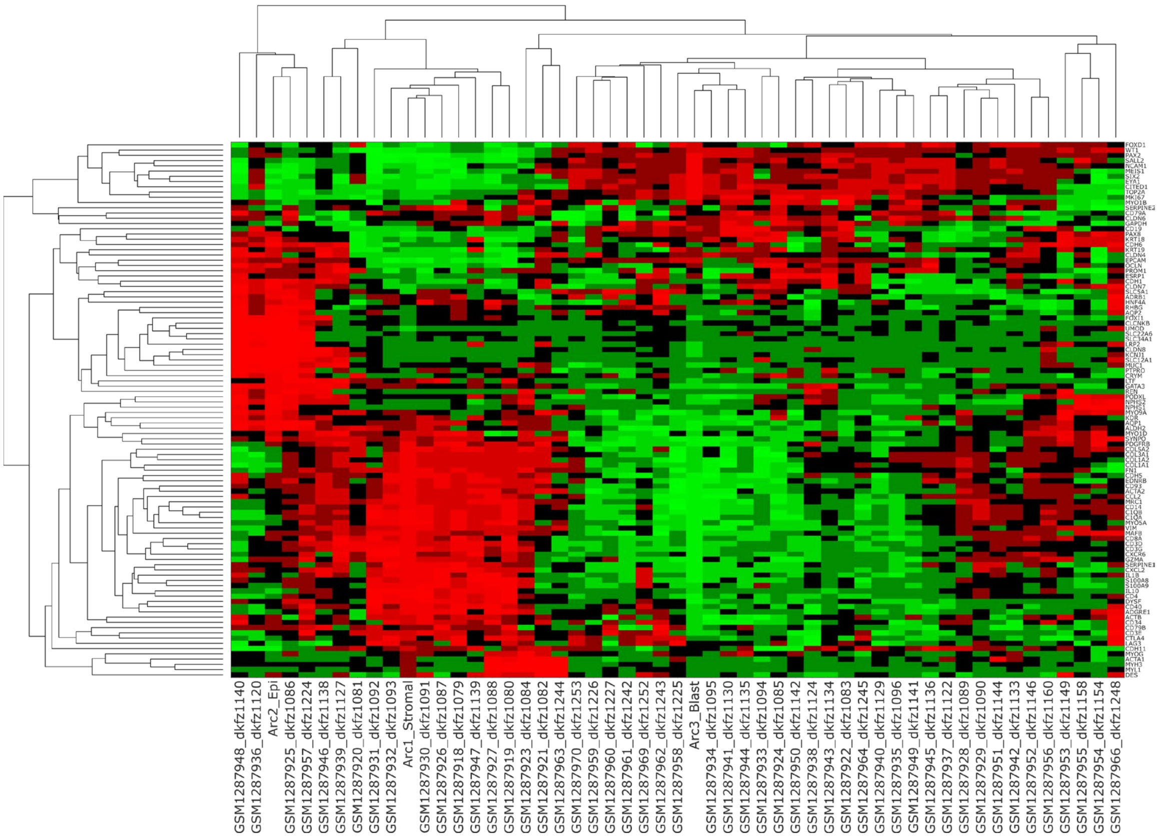

Figure S12: A heatmap of selected genes vs. tumors and archetypes showing the **sample ID** (GEO accession number and DKFZ identifier).

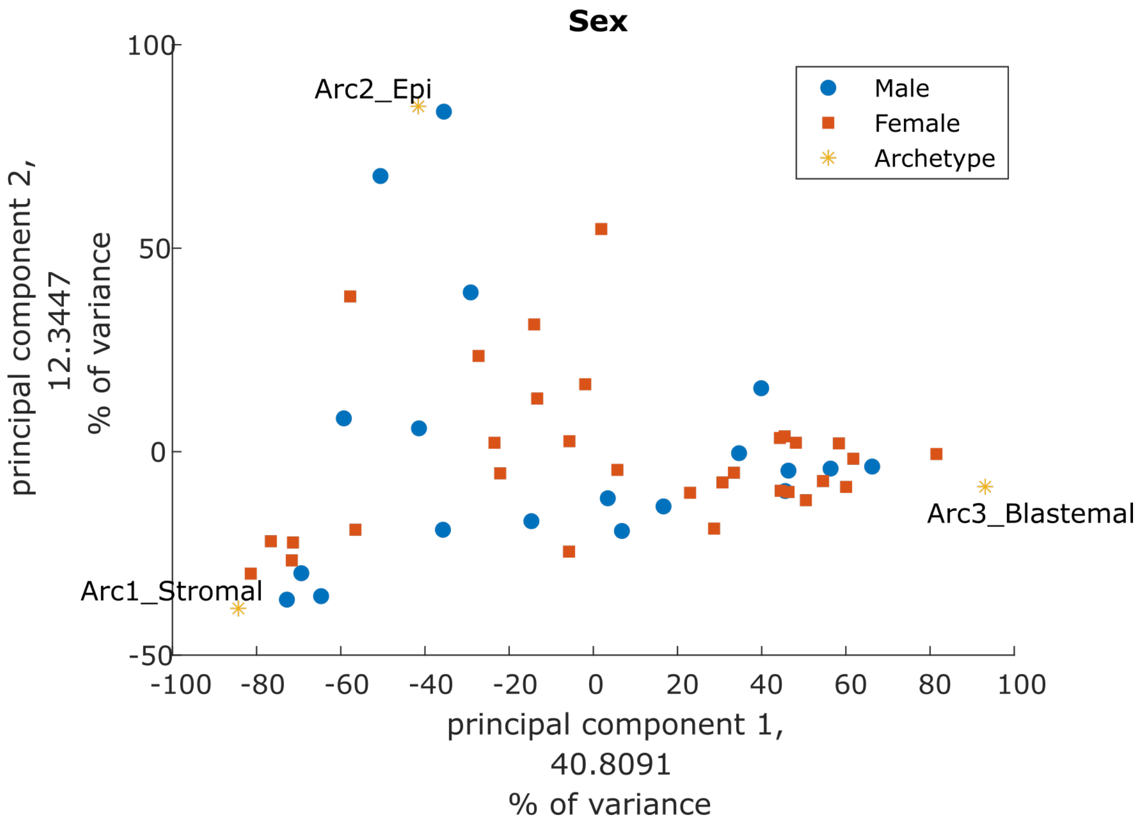

Figure S13: PCA of tumors and archetypes showing the reported **patient sex** (male/female).

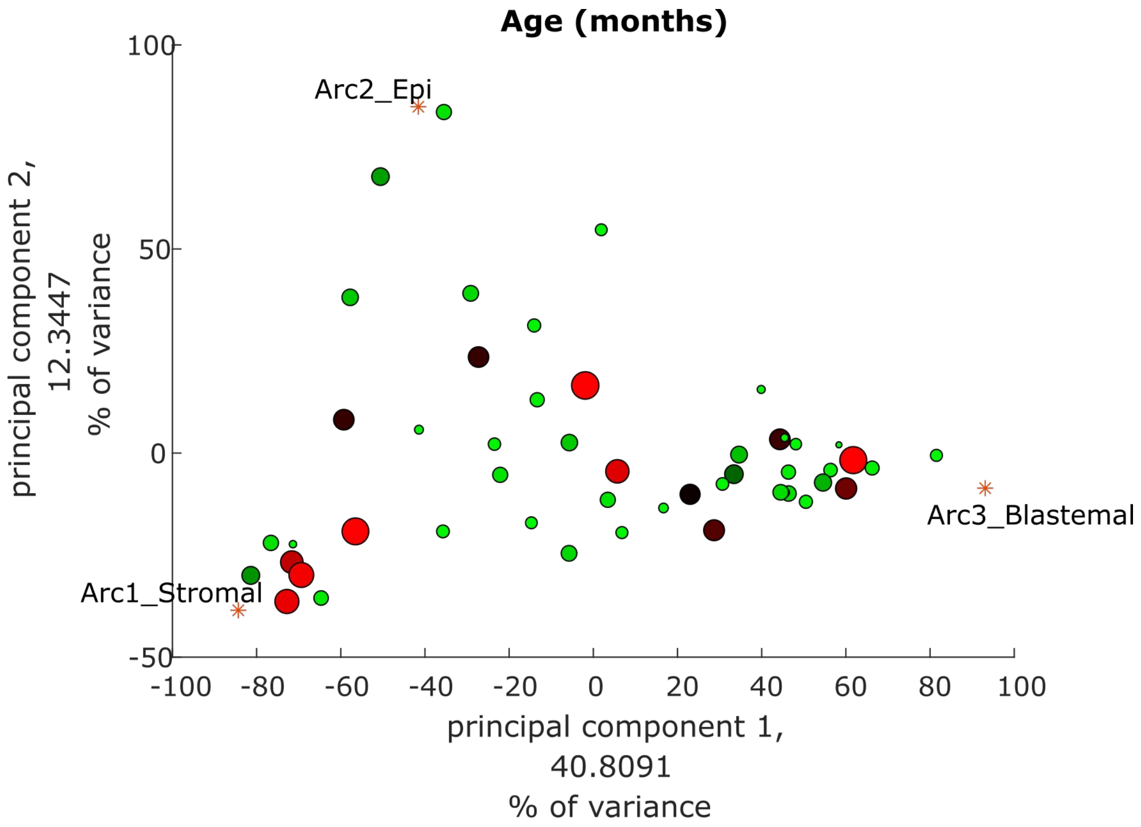

Figure S14: PCA of tumors and archetypes showing **patient age** (in months) at diagnosis (old – red/large symbol, young – green/small symbol).

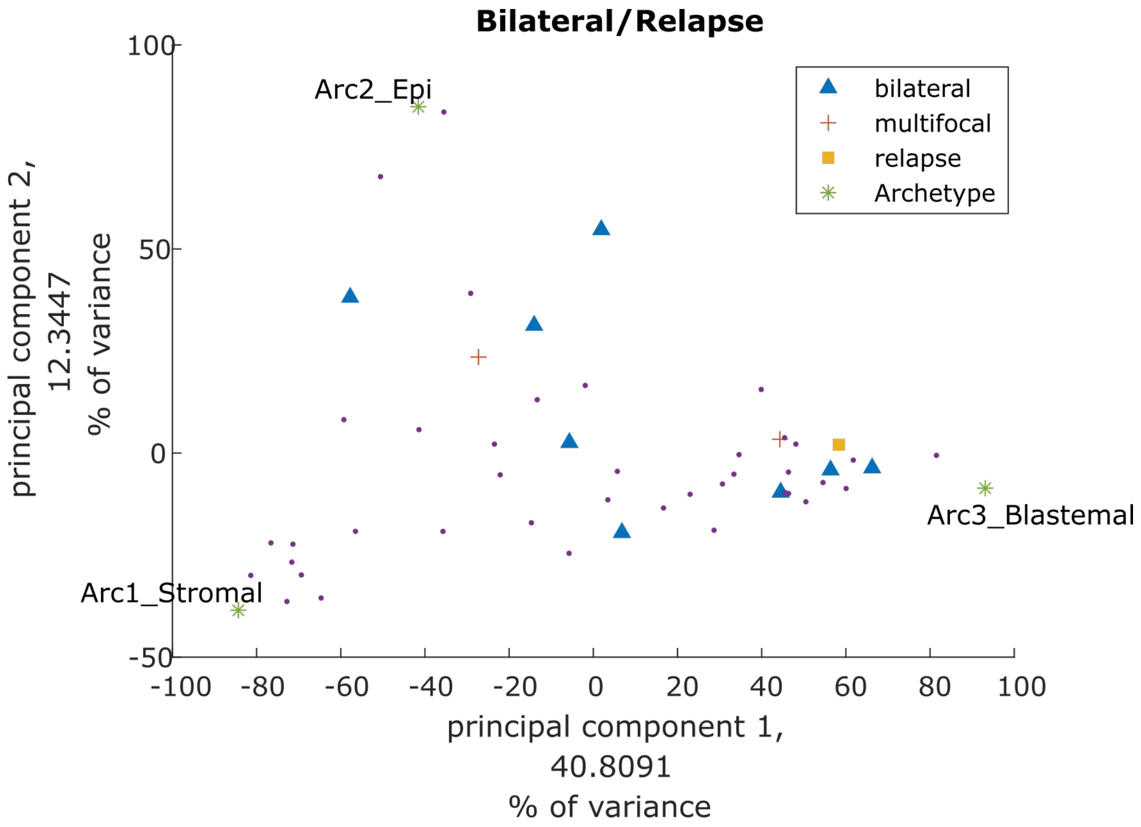

Figure S15: PCA of tumors and archetypes showing the reported clinical data (**bilateral and/or relapse**). It can be seen that bilateral tumors tend to cluster away from the stromal archetype.

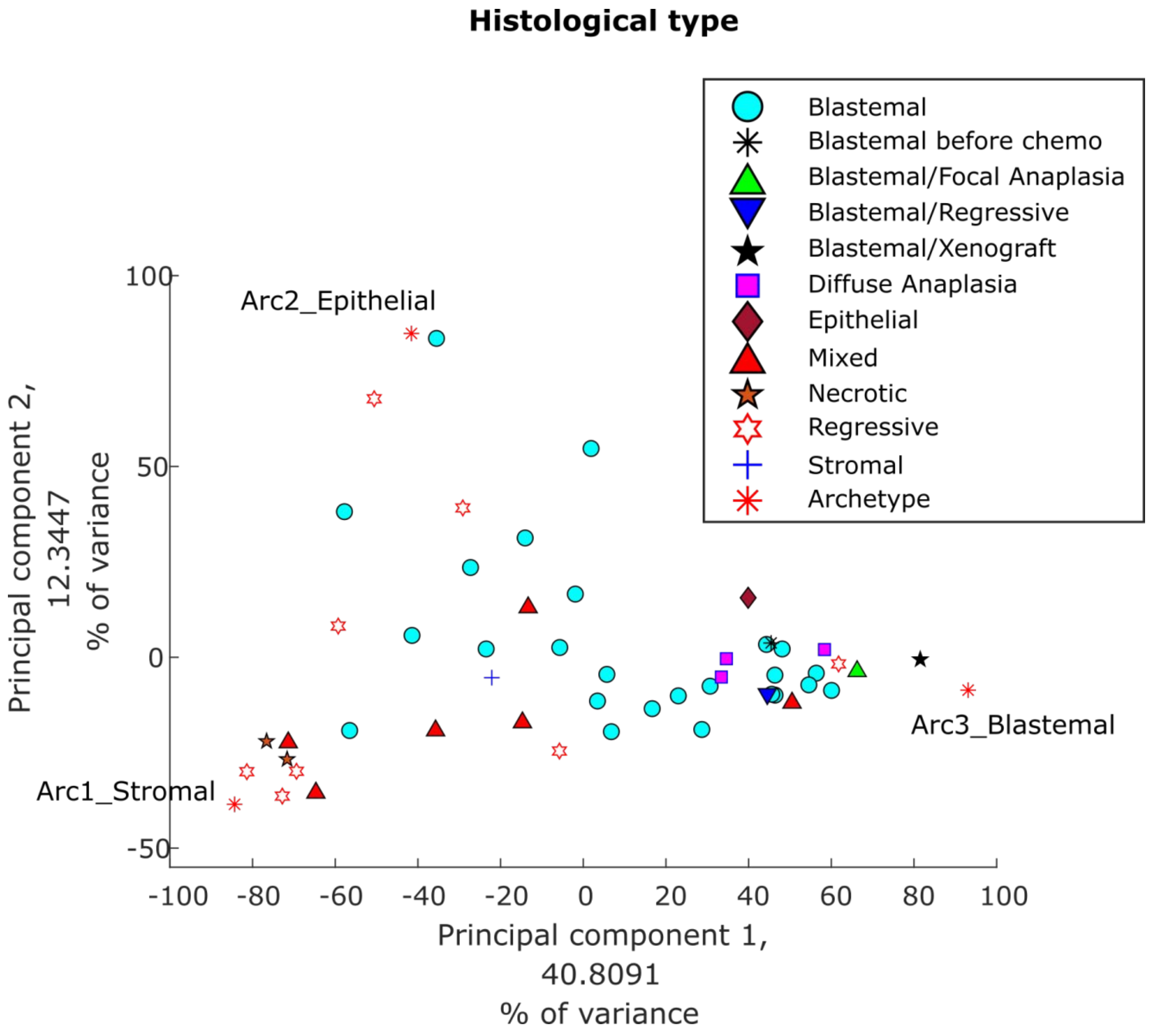

Figure S16: PCA of tumors and archetypes showing the reported **histological type**. It can be seen that the tumors with anaplastic histology (diffuse or focal), which is considered least favorable, tend to cluster in the vicinity of the blastemal archetype. Likewise, notice that the single blastema-only xenograft in the dataset is located closest to the blastemal archetype, more than any other tumor. This is consistent with previous observations that patient-derived xenografts significantly increase the percentage of their blastemal component from their first passage [1].

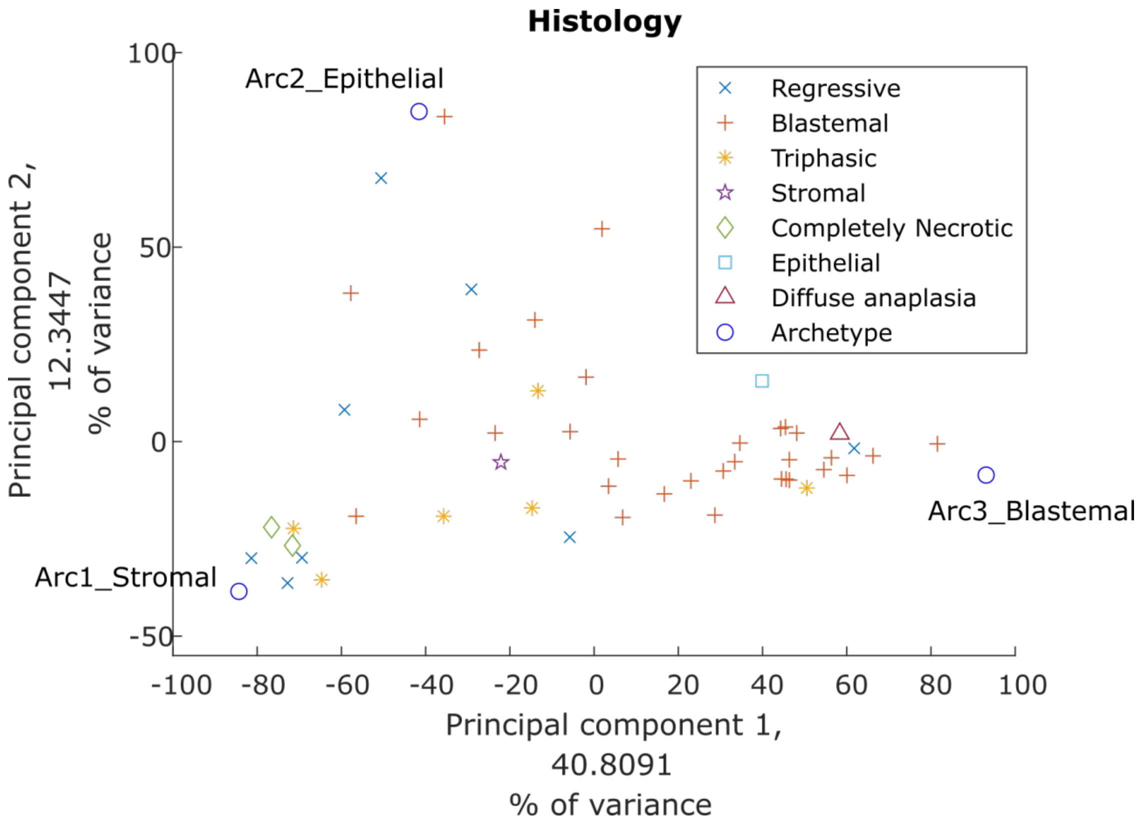

Figure S17: PCA of tumors and archetypes showing the reported tumor **histology**.

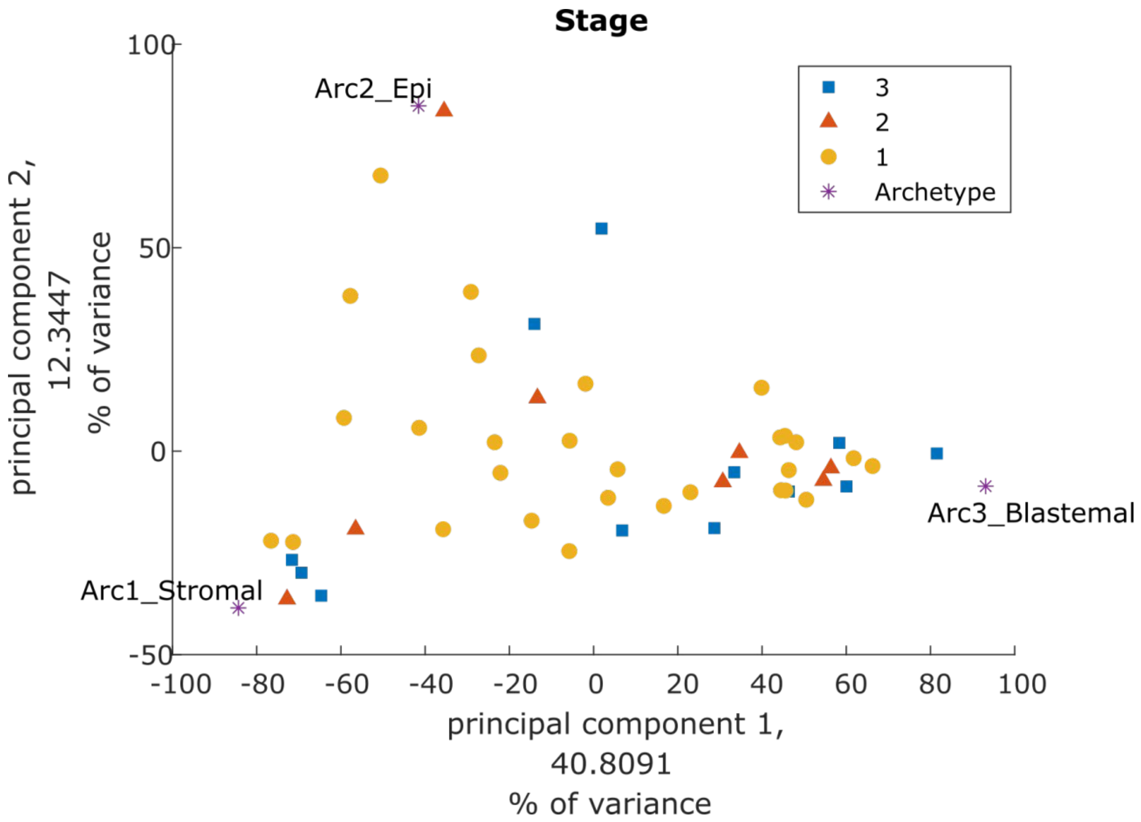

Figure S18: PCA of tumors and archetypes showing the reported tumor **stage** at diagnosis (a single tumor with unknown stage was not plotted).

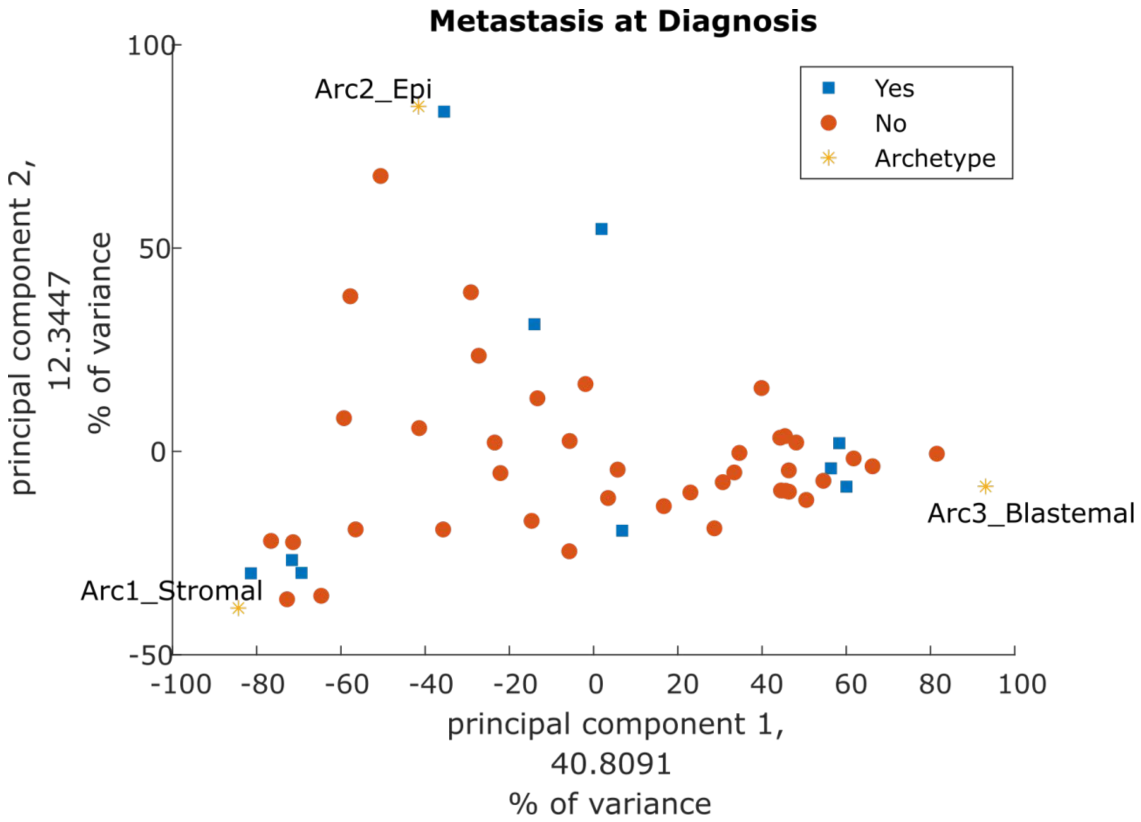

Figure S19: PCA of tumors and archetypes showing the reported tumor **metastasis at diagnosis**.

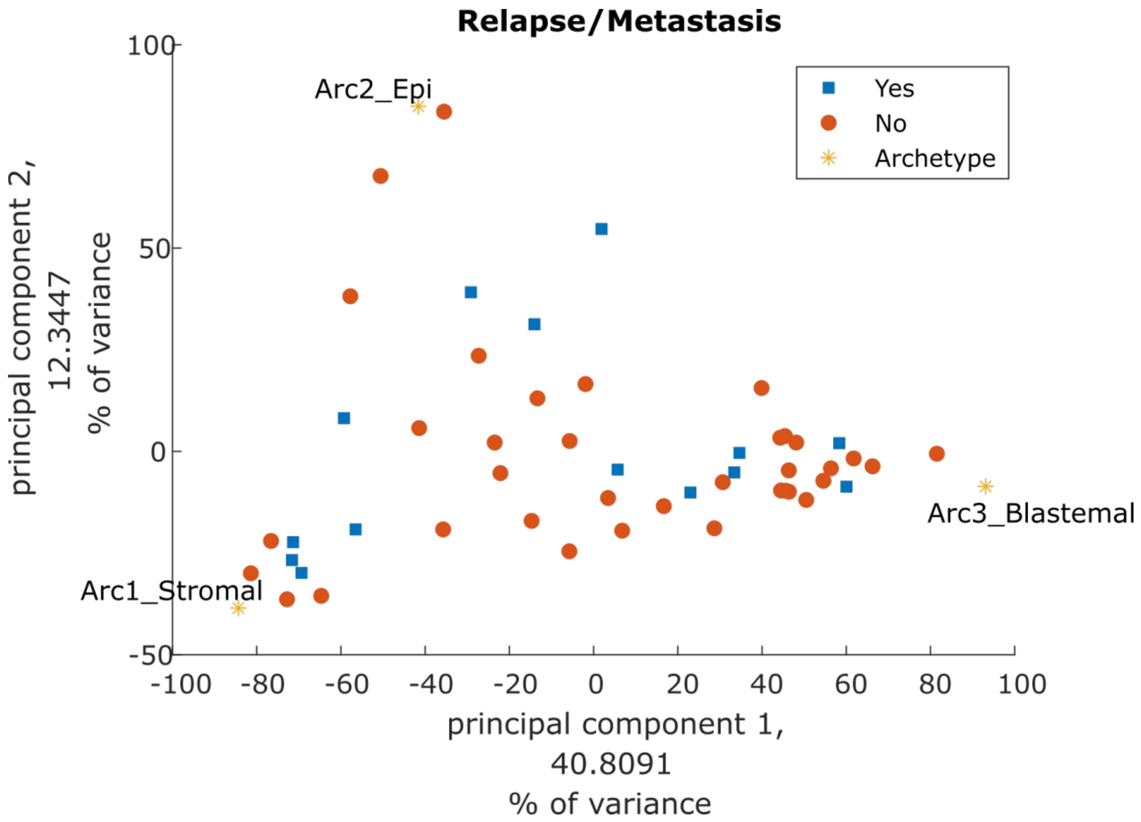

Figure S20: PCA of tumors and archetypes showing the reported status of the tumor (relapse/metastasis).

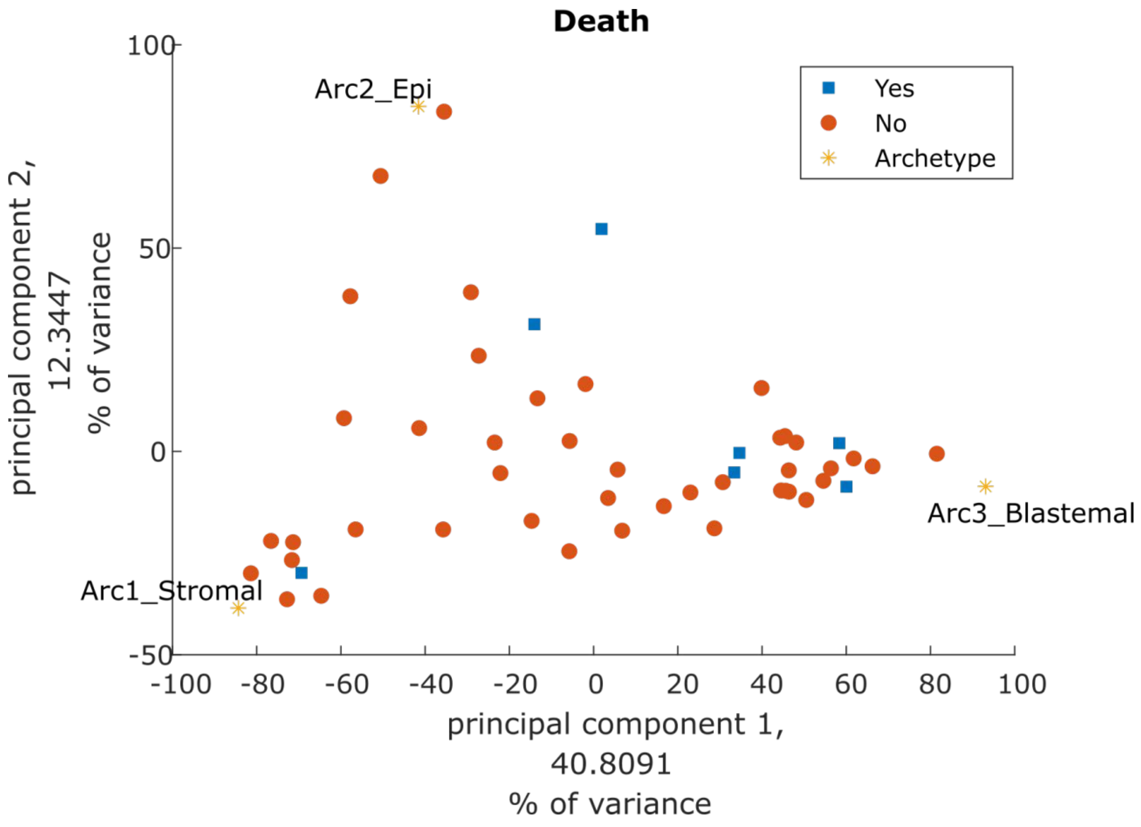

Figure S21: PCA of tumors and archetypes showing the reported status of the patient (**death**).

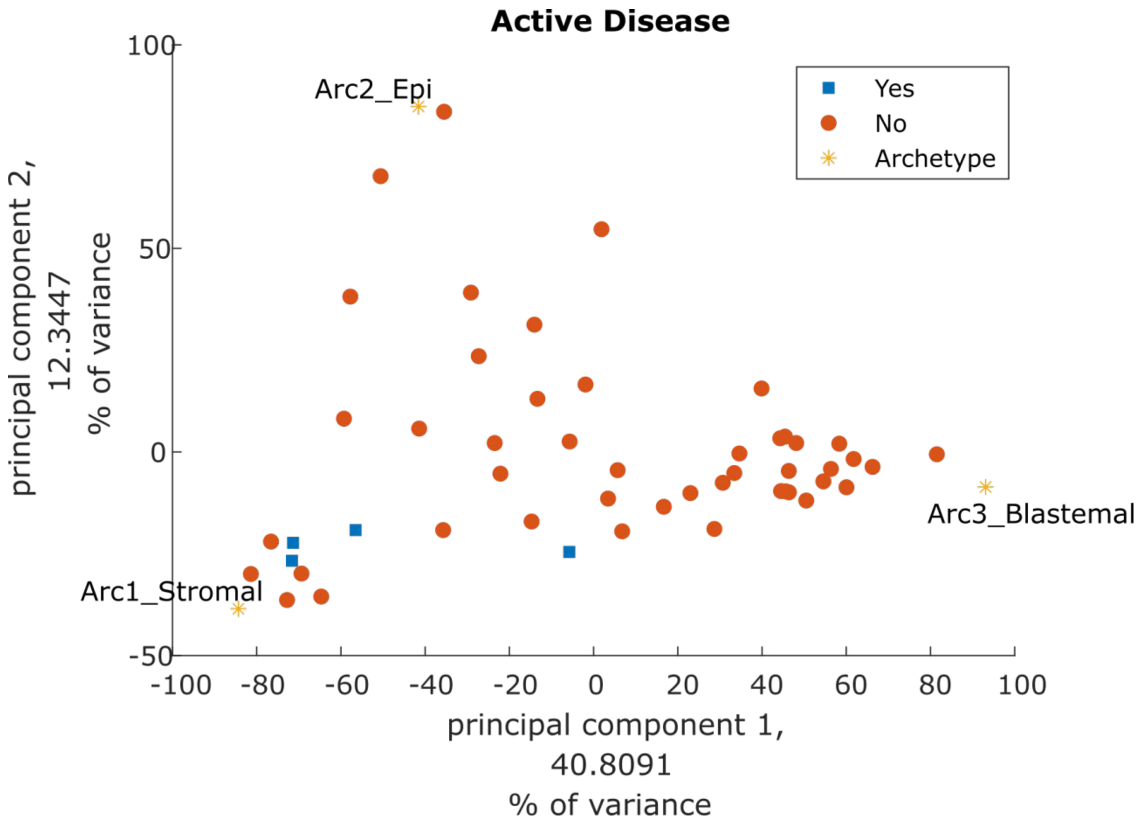

Figure S22: PCA of tumors and archetypes showing the reported activity of the tumor (**active disease**). It can be seen that most tumors from patients with active disease in this dataset tend to locate near the stromal archetype.

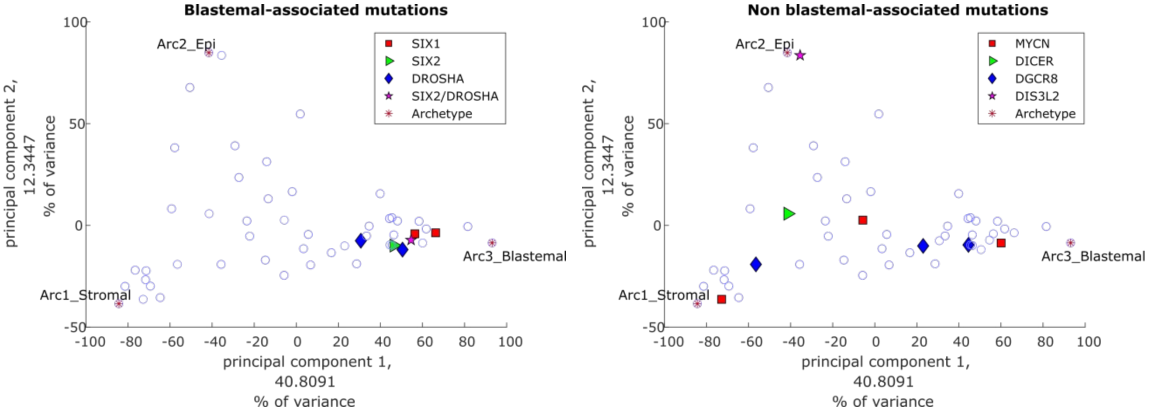

Figure S23: PCA of tumors and archetypes showing the reported **mutations**. It can be seen that mutations in the genes SIX1, SIX2, or DROSHA (left panel) tend to cluster in the vicinity of the blastemal archetype. This agrees with the higher incidence of SIX1/2 mutations in tumors with chemotherapy-resistant blastema that was observed by Wegert et al. (Wegert et al., 2015).

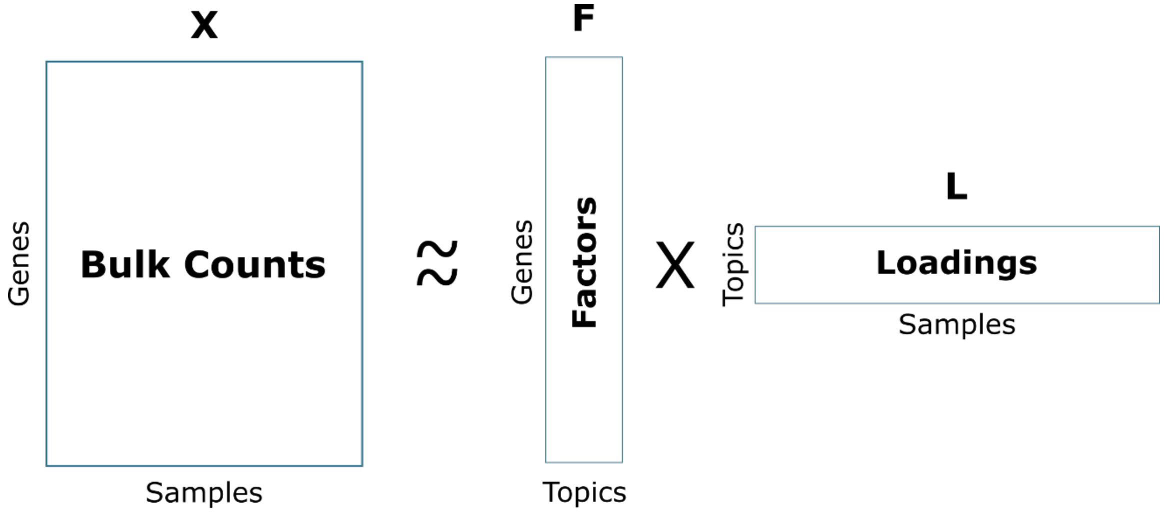

$$X \approx F \times L$$

Figure S24: A sketch of non-negative matrix factorization (NMF) that is used to fit the topic model in this study (see Carbonetto *et al.* [2] for more details). The matrix X, consisting of bulk gene expression counts, is factorized using non-negative matrix factorization into a “Factors” matrix F and a “Loadings” matrix L. The columns of the “Factors” matrix F represent $p(gene|topic)$ - the probability of a transcript from a given gene being expressed in each one of the k topics. The rows of the “Loadings” matrix L represent $p(topic|sample)$, which are the proportions $\theta_1, \theta_2, \dots, \theta_k$ that each of the k topics contribute to each bulk tumor sample. In the “fastTopics” R package that was used in our study the parameters of the topic model are learned by performing 100 iterations of the expectation maximization (EM) algorithm followed by 100 iterations of the coordinate descent (CD) algorithm.

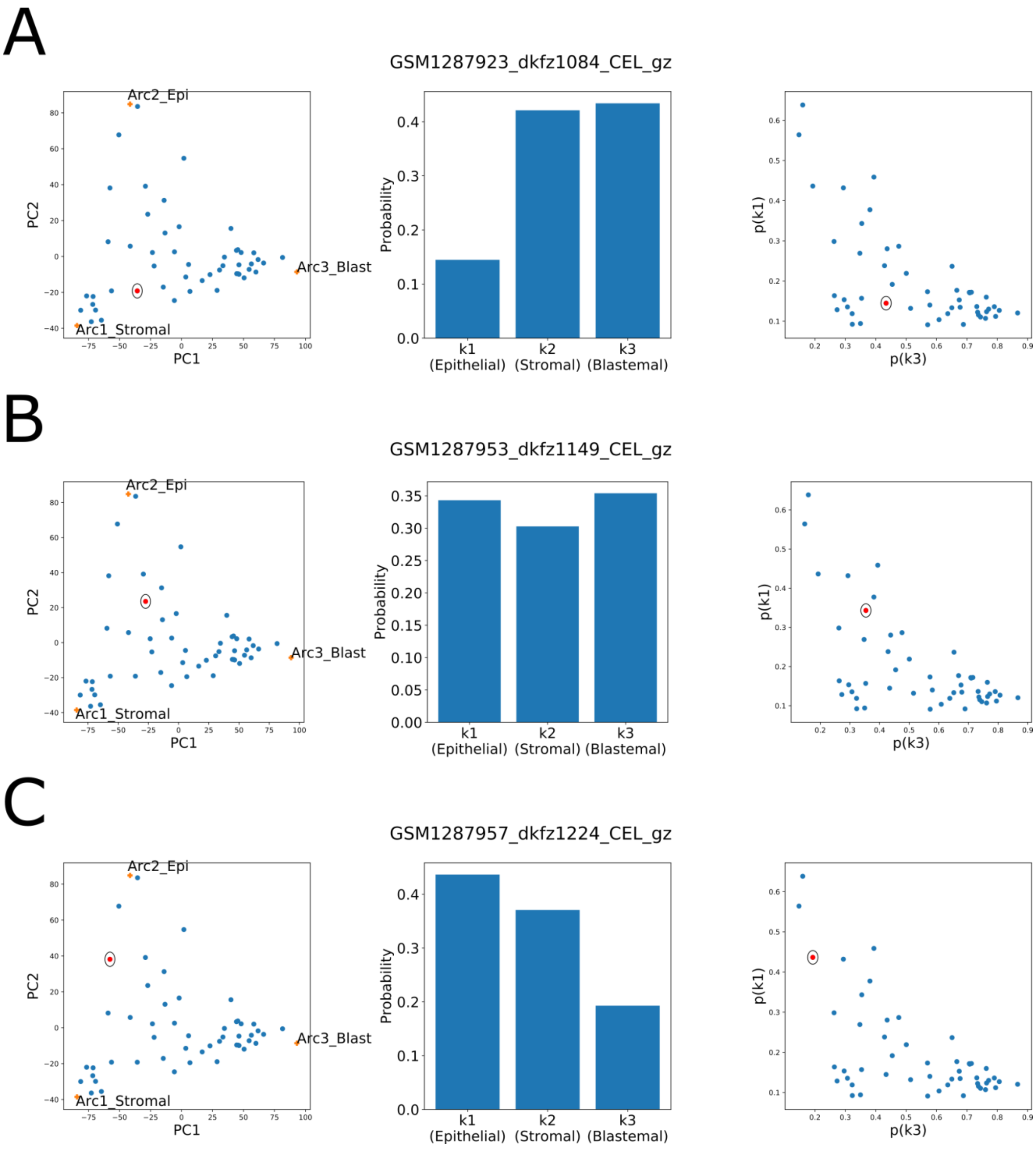

Figure S25: LDA topic modeling shows that tumors in-between the vertexes of the triangle-shaped continuum (left panels) are composed of multiple topics (middle panels), in consistency with the topic simplex (right panels).

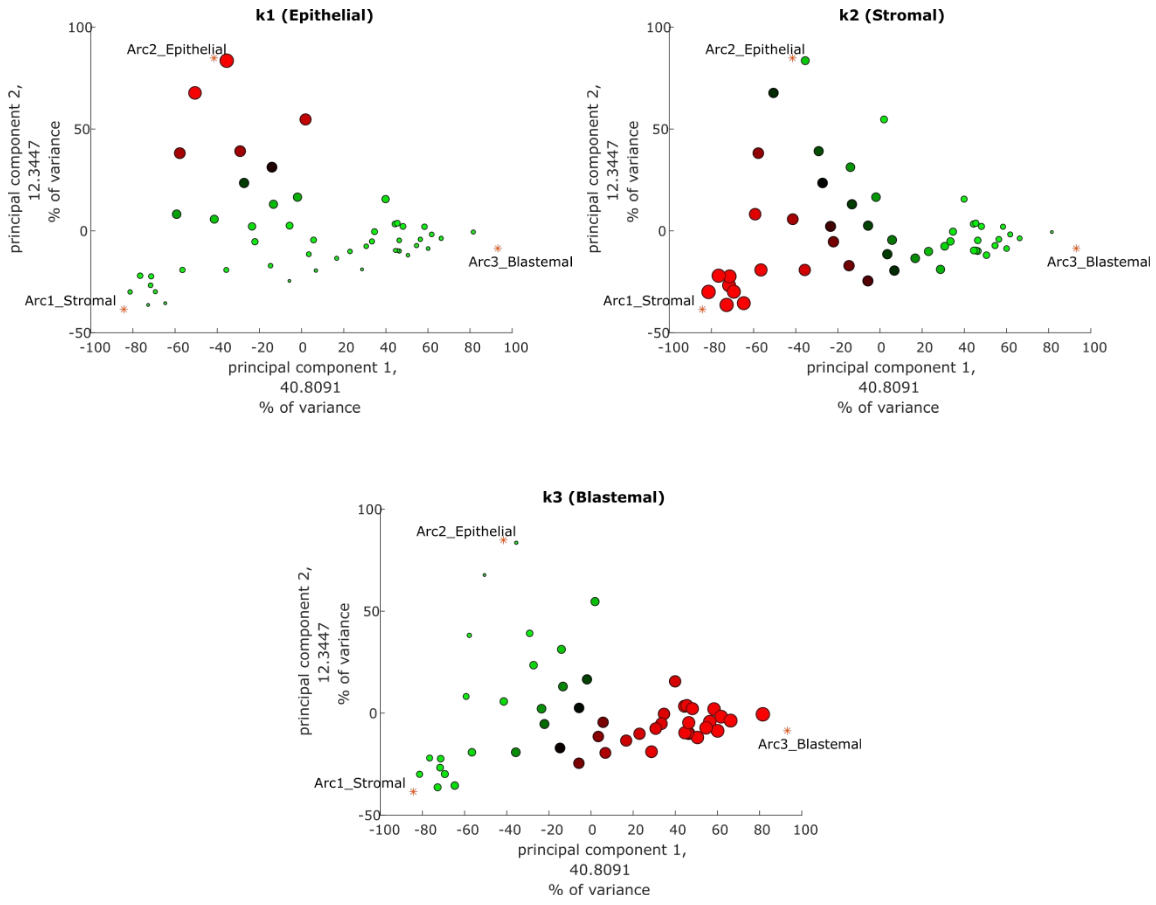

Figure S26: The proportions of each of the three topics are highest towards each of the different vertexes of the triangle-shaped continuum. Shown are PCA plots of the tumors in our dataset. For each topic, large proportion – red/large symbol, small proportion – green/small symbol.

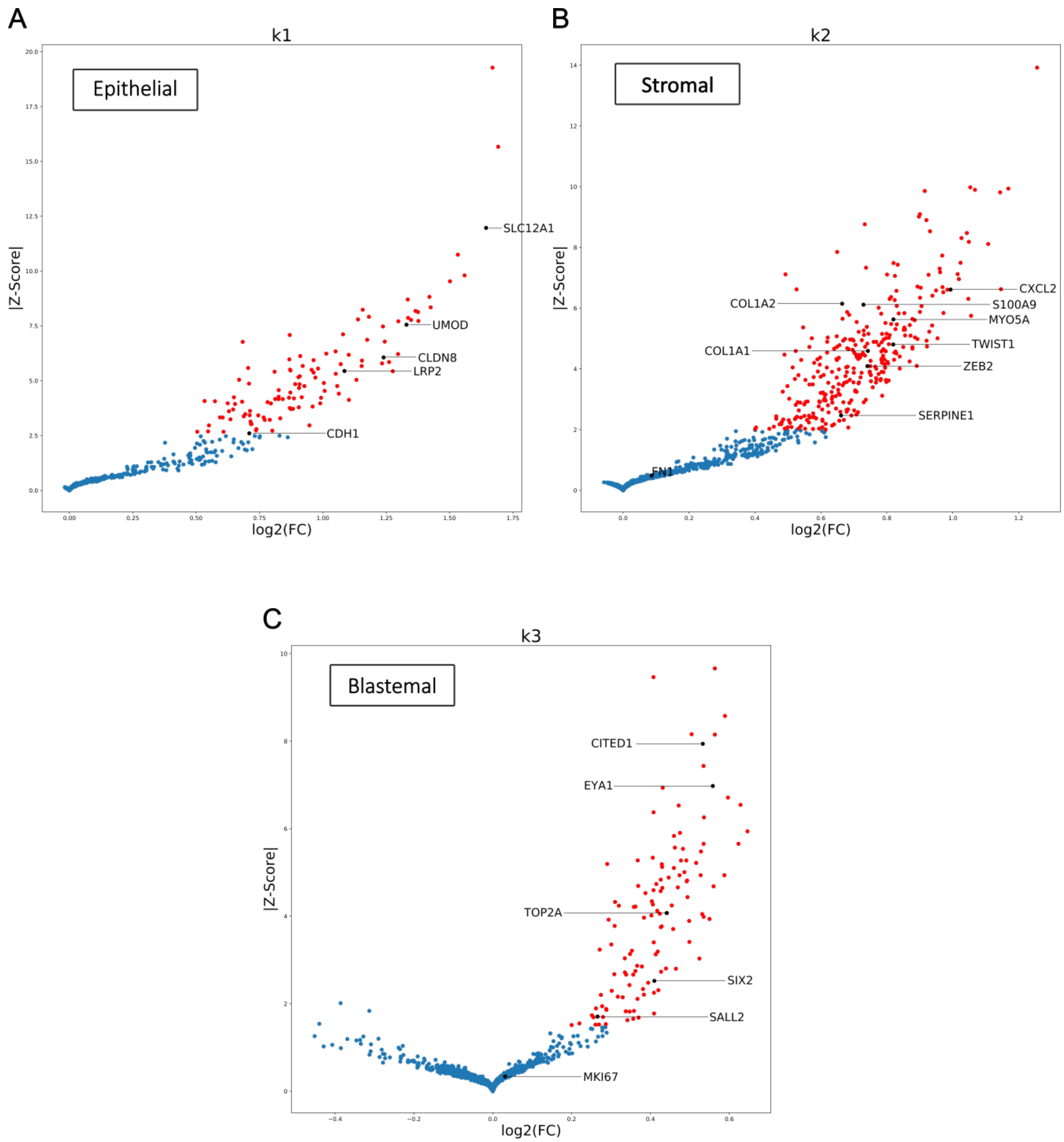

Figure S27: The three topics over-express genes related to renal epithelial, stromal, or blastemal characteristics. Shown are volcano plots of the log-fold-change vs Z-score for each of the topics (compared against the null model). We marked genes that are known to be over-expressed in the renal epithelial structures, the un-induced mesenchyme (stroma), and the Cap mesenchyme (blastema) of the fetal kidney.

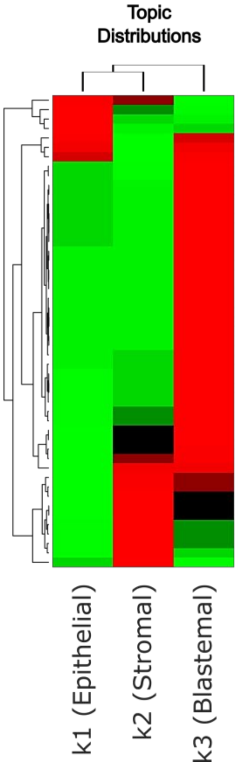

Sample names	Sex (male/female)	Age (months)	bilateral/Relapse	Histological Type	Histology	Stage	Metastasis at Diagnosis	Relapse/Metastasis	Death	Active Disease	Mutations
GSM1287957	Female	50	Bilateral	Blastemal	Blastemal	1					
GSM1287925	Male	58		Regressive	Regressive	1					
GSM1287948	Male	41		Blastemal	Blastemal	2	y				DIS3L2 c.C2479T:p.Q827X * (het)
GSM1287966	Male	45	-	Regressive	Regressive	1		y			
GSM1287936	Female	22	Bilateral	Blastemal	Blastemal	3	y	y	y		
GSM1287954	Female	29	Bilateral	Blastemal	Blastemal	3	y	y	y		
GSM1287953	Female	82	Multifocal	Blastemal	Blastemal	1					
GSM1287933	Male	6		Epithelial	Epithelial type	1					
GSM1287955	Female	153	-	Blastemal	Blastemal	1					
GSM1287958	Female	23	-	Blastemal/xenograft	Blastemal	3					
GSM1287924	Female	NaN	Relapse	Diffuse anaplasia	Diffuse anaplasia	3	y	y	y		
GSM1287962	Female	20	-	Blastemal	Blastemal	1					
GSM1287959	Female	83	Multifocal	Blastemal	Blastemal	1					
GSM1287934	Female	148		Regressive	Regressive	1					
GSM1287960	Female	5	-	Blastemal before chemotherapy	Blastemal	1					
GSM1287941	Male	34	Bilateral	Blastemal/focal anaplasia	Blastemal	1					SIX1 c.A530G, p.Q177R (hom)
GSM1287944	Male	31	Bilateral	Blastemal	Blastemal	2	y				SIX1 c.A530G, p.Q177R (het)
GSM1287938	Male	52	-	Diffuse anaplasia	Blastemal	2		y	y		
GSM1287950	Female	88		Blastemal	Blastemal	3	y	y	y		MYCN c.C130A:p.P44T & c.C131T:p.P44L (hom)
GSM1287961	Female	55		Blastemal	Blastemal	2					SIX2 c.A530G, p.Q177R (het); DROSHA c.G3439A:p.E1147K (het)
GSM1287964	Male	35	-	Blastemal	Blastemal	1					
GSM1287921	Female	35		Mixed	Triphasic	2					
GSM1287922	Female	43		Blastemal	Blastemal	3					SIX2 c.A530G, p.Q177R (het)
GSM1287943	Female	66	-	Diffuse anaplasia	Blastemal	3		y	y		
GSM1287935	Female	29		Mixed	Triphasic	1					DROSHA c.G3439A:p.E1147K (het)
GSM1287949	Female	44	Bilateral	Blastemal/regressive	Blastemal	1					DGCR8 c.G1552A:p.E518K (hom)
GSM1287940	Male	8	-	Blastemal	Blastemal	1					
GSM1287970	Female	26		Blastemal	Blastemal	2					DROSHA c.G3664A:p.E1222K (het)
GSM1287945	Female	78		Blastemal	Blastemal	1		y			DGCR8 c.243_244insT:p.K82X (hom)
GSM1287963	Female	109	-	Blastemal	Blastemal	1		y			
GSM1287969	Female	85	-	Blastemal	Blastemal	3					
GSM1287952	Female	50	Bilateral	Blastemal	Blastemal	1					MYCN c.C131T:p.P44L (het)
GSM1287937	Male	12	-	Blastemal	Blastemal	1					
GSM1287942	Male	41		Blastemal	Blastemal	1					
GSM1287951	Male	23	Bilateral	Blastemal	Blastemal	3	y				
GSM1287956	Female	25	-	Blastemal	Blastemal	1					
GSM1287929	Female	45		Regressive	Regressive	1				y	
GSM1287928	Male	22		Mixed	Triphasic	1					
GSM1287920	Female	41		Stromal	Stromal type	1					
GSM1287923	Male	27		Mixed	Triphasic	1					
GSM1287946	Male	9		Blastemal	Blastemal	1					DICER1 c.G5656A:p.G1886R * (het)
GSM1287947	Female	146		Blastemal	Blastemal	2		y		y	DGCR8 c.G637T:p.E213X (het)
GSM1287927	Male	37		Mixed	Triphasic	3					
GSM1287932	Male	116		Regressive	Regressive	2					MYCN c.C131T:p.P44L (het)
GSM1287919	Male	124		Regressive	Regressive	3	y	y	y		
GSM1287918	Female	101		Necrotic	Completely necrotic	3	y	y		y	
GSM1287926	Female	4		Mixed	Triphasic	1		y		y	
GSM1287930	Female	60		Regressive	Regressive	-	y				
GSM1287931	Female	42		Necrotic	Completely necrotic	1					
GSM1287939	Male	82	-	Regressive	Regressive	1		y			

Figure S28: A heatmap showing the topic distribution of each tumor vs. its histological and clinical metadata. It can be seen that tumors with anaplastic histology (diffuse or focal), which is considered least favorable, as well as the single blastemal xenograft in the dataset, and tumors with mutations in the genes SIX1, SIX2, or DROSHA, all contain a large fraction of the blastemal topic.

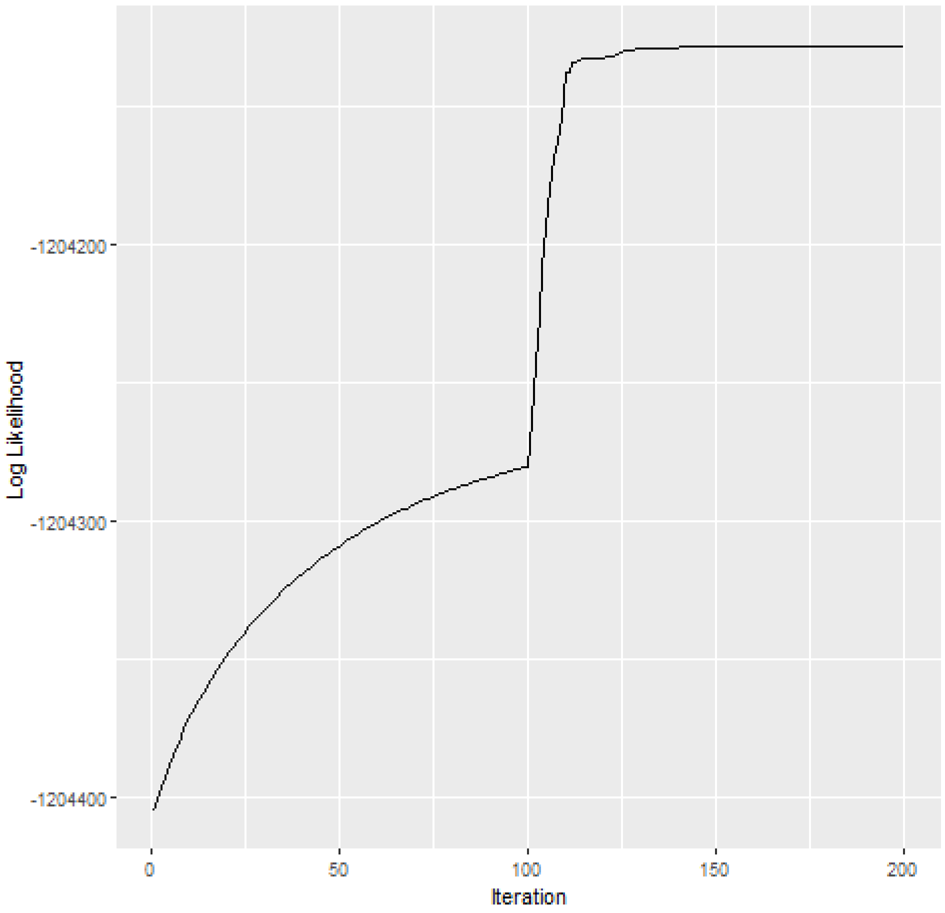

Figure S29: The log-likelihood score of the non-negative matrix factorization (NMF) algorithm that was used for fitting a topic model with k=3 topics to the “bulk" gene expression matrix. Following Carbonetto *et al.* [2], the “fastTopics” R package that we used learns the parameters of the topic model by performing 100 iterations of the expectation maximization (EM) algorithm followed by 100 iterations of the coordinate descent (CD) algorithm.

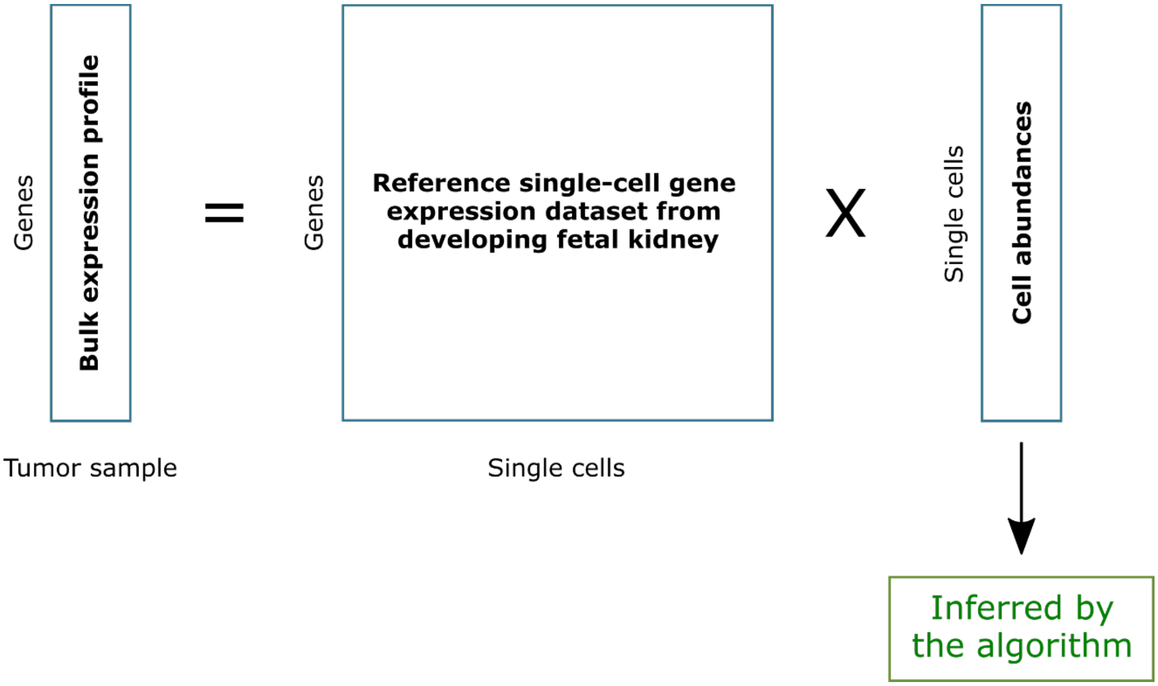

Figure S30: A sketch of the cell deconvolution procedure. The algorithm accepts the "bulk" gene expression profiles of tumors and a reference single-cell gene expression matrix as input and uses support vector regression (SVR) to infer the cell abundances in each tumor. In the CPM algorithm used in this study (see Frishberg *et al.* [3] for details), SVR is repeatedly performed on subsets of cells that are randomly selected from the reference single-cell expression matrix. The procedure is repeated such that each cell is selected at least a minimal number of times ("minSelection") predefined by the user and the results are averaged for each cell. The population proportions are inferred using the single cell abundance values summed up over all cells from the same population.

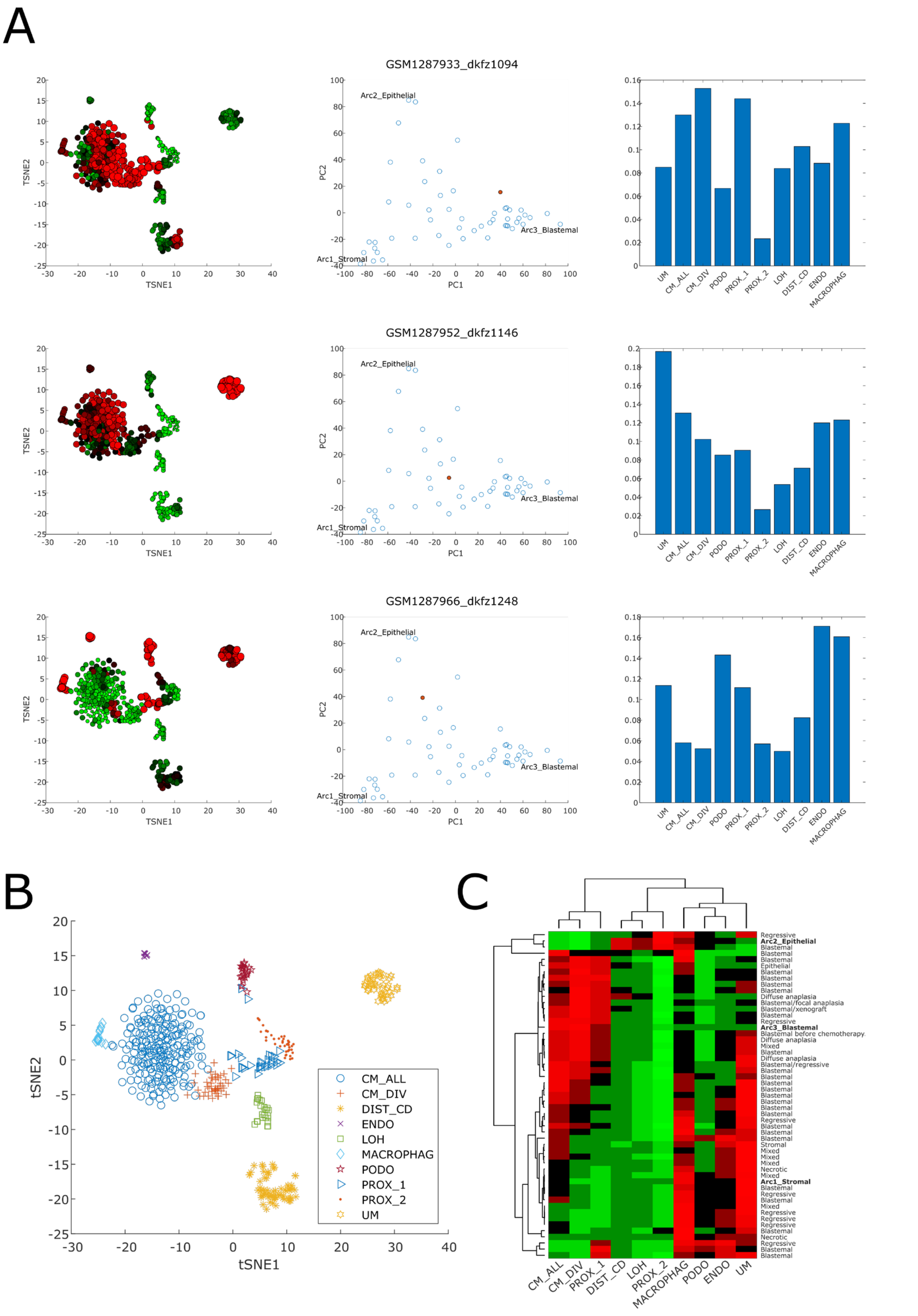

Figure S31: Cellular deconvolution indicates that each tumor is composed of a unique mixture of cell populations resembling those of the fetal kidney. (A) Shown is a cellular

deconvolution of selected tumors located within the triangle-shaped continuum that is spanned by the stromal, blastemal, and epithelial archetypes. It can be seen that tumors located in-between the three archetypes are composed of a mixture of heterogeneous cell types, for example, cells resembling the Cap mesenchyme and the renal epithelium (top panel), cells resembling the Cap mesenchyme and the un-induced mesenchyme (middle panel), and cells resembling the un-induced mesenchyme and the renal epithelium (with only a minority of cells resembling the Cap mesenchyme, bottom panel). This is in contrast to the archetypes that are each predominantly composed of a single cell type (resembling the un-induced mesenchyme, the Cap-mesenchyme, or epithelial cells, see Fig. 4). (B) The different cell populations marked on a tSNE plot of the reference single cell RNAseq dataset from the developing mouse fetal kidney that was used for cellular deconvolution (CM – Cap mesenchyme, DIST_CD – distal tubule and collecting duct, ENDO – endothelial, LOH – Loop of Henle, MACROPHAG – macrophages, PODO – podocytes, PROX_1 – early epithelial structures such as C/S-shaped bodies, PROX_2 – proximal tubule, UM – un-induced mesenchyme). (C) A heatmap of the cell type proportions from which each tumor is composed, as predicted by cellular deconvolution.

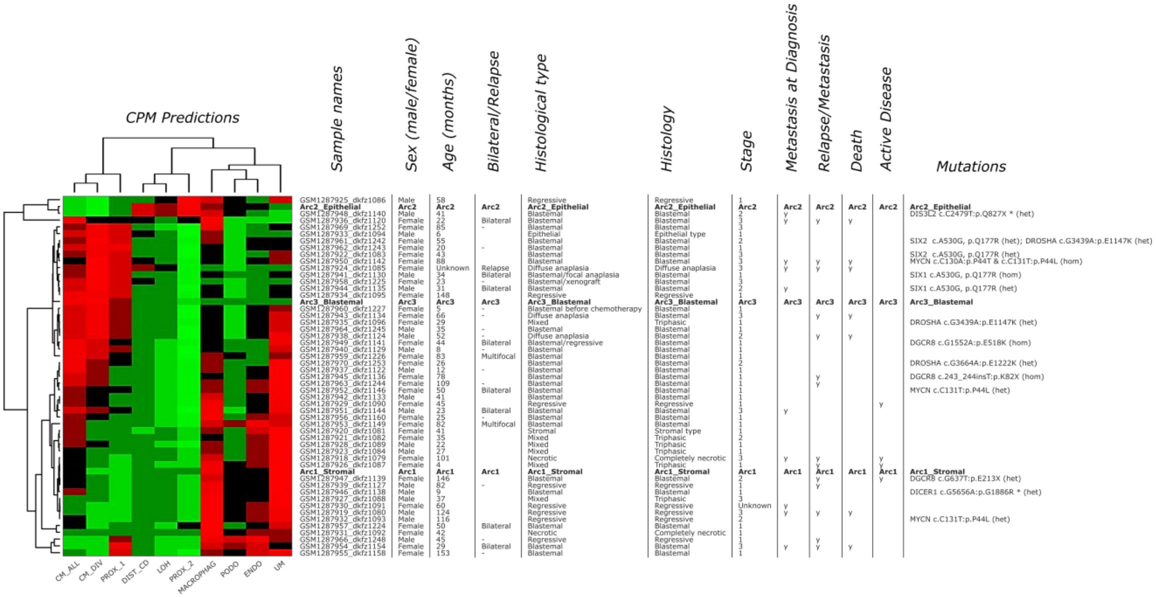

Figure S32: A heatmap of the cell type proportions from which each tumor is composed, as predicted by cellular deconvolution, along with tumor histological and clinical metadata. It can be seen that most of the tumors with reported blastemal histology contain a significant proportion of cells resembling those of the Cap mesenchyme (CM_ALL), as expected. Likewise, it can be seen that tumors with anaplastic histology (diffuse or focal), as well as the single blastemal xenograft in our dataset, and also tumors reported to contain mutations in the genes SIX1, SIX2, or DROSHA, contain a significant fraction of cells resembling the cycling Cap mesenchyme cells (CM_DIV) in the fetal kidney. This is in agreement with the findings of Wegert *et al*. [4] that blastemal type Wilms tumors with mutations in SIX1 or SIX2 have a have a gene expression signature of proliferation and kidney progenitors. On the other hand, it can be seen that tumors reported to have triphasic/mixed or regressive histology contain high proportions of cells resembling the un-induced mesenchyme (UM).

REFERENCES

1. Murphy AJ, Chen X, Pinto EM, Williams JS, Clay MR, Pounds SB, et al. Forty-five patient-derived xenografts capture the clinical and biological heterogeneity of Wilms tumor. Nature communications. 2019;10: 5806. doi:10.1038/s41467-019-13646-9

2. Carbonetto P, Sarkar A, Wang Z, Stephens M. Non-negative matrix factorization algorithms greatly improve topic model fits. arXiv; 2022. Available: http://arxiv.org/abs/2105.13440

3. Frishberg A, Peshes-Yaloz N, Cohn O, Rosentul D, Steuerman Y, Valadarsky L, et al. Cell composition analysis of bulk genomics using single-cell data. Nature Methods. 2019;16. doi:10.1038/s41592-019-0355-5

4. Wegert J, Ishaque N, Vardapour R, Ge??rg C, Gu Z, Bieg M, et al. Mutations in the SIX1/2 Pathway and the DROSHA/DGCR8 miRNA Microprocessor Complex Underlie High-Risk Blastemal Type Wilms Tumors. Cancer Cell. 2015;27: 298–311. doi:10.1016/j.ccell.2015.01.002
