# Supplementary material for: Characterization of Continuous Transcriptional Heterogeneity in High-Risk Blastemal-Type Wilms’ Tumors Using Unsupervised Machine Learning
Source: Int J Mol Sci. 2023 Feb 9;24(4):3532. doi: 10.3390/ijms24043532 (PMC9965420; doi:10.3390/ijms24043532)
Supplement: Supplementary file 1 [file ijms-24-03532-s001.zip › ijms-2132028-supplementary tables legend.pdf]

Table S1: Normalized gene expression values of the Wilms' tumors from Wegert et al. and the three archetypes.

Table S2: Integrated histological and clinical data from Wegert et al., including the mapping from the sample IDs of the mRNA microarrays (WT055, WT0056….) to the sample IDs in the GEO database (dkfz1079, dkfz1080….)

Table S3: A list of 102 selected genes that are known from the literature to be associated with kidney development and tumorigenesis.

Table S4: Gene Ontology (GO) enrichment analysis for the *stromal* archetype. Genes for which log2FC > 2 in the *stromal* archetype with respect to the other two archetypes were selected and inserted to Toppgene.

Table S5: Gene Ontology (GO) enrichment analysis for the *epithelial* archetype. Genes for which log2FC > 2 in the *epithelial* archetype with respect to the other two archetypes were selected and inserted to Toppgene.

Table S6: Gene Ontology (GO) enrichment analysis for the *blastemal* archetype. Genes for which log2FC > 2 in the *blastemal* archetype with respect to the other two archetypes were selected and inserted to Toppgene.

Table S7: Gene Ontology (GO) enrichment analysis for the *epithelial* topic (k1). Genes for which the posterior probabilities $p(topic = k1|gene) > 1/2$ were selected and inserted to Toppgene.

Table S8: Gene Ontology (GO) enrichment analysis for the *stromal* topic (k2). Genes for which the posterior probabilities $p(topic = k2|gene) > 1/2$ were selected and inserted to Toppgene.

Table S9: Gene Ontology (GO) enrichment analysis for the *blastemal* topic (k3). Genes for which the posterior probabilities $p(topic = k3|gene) > 1/2$ were selected and inserted to Toppgene. Program: A compressed directory containing programs and datasets for data visualization, and well as sets of figures characterizing each tumor
